# The Factors Affecting the Stability of IOP Homeostasis

**DOI:** 10.1167/iovs.65.6.4

**Published:** 2024-06-04

**Authors:** Darryl R. Overby, C. Ross Ethier, Changxu Miao, Ruth A. Kelly, Ester Reina-Torres, W. Daniel Stamer

**Affiliations:** 1 Department of Bioengineering, Imperial College London, London, United Kingdom; 2 Wallace H. Coulter Department of Biomedical Engineering at Georgia Institute of Technology & Emory University School of Medicine, Atlanta, Georgia, United States; 3 Department of Ophthalmology, Duke University Medical School, Durham, North Carolina, United States

**Keywords:** trabecular meshwork, aqueous humor outflow, mathematical modelling, mechanobiology, outflow facility measurements

## Abstract

**Purpose:**

Shear-induced nitric oxide (NO) production by Schlemm's canal (SC) endothelial cells provides a fast, IOP-sensitive feedback signal that normally contributes to IOP homeostasis. Our goal was to analyze the response of this homeostatic system under constant flow perfusion (as occurs in vivo) vs. constant pressure perfusion (as typical for laboratory perfusions).

**Methods:**

A mathematical model of aqueous humor dynamics, including shear-mediated NO signaling, was formulated and analyzed for stability. The model includes Goldmann's equation, accounting for proximal and distal outflow resistance, and describes how elevated IOP causes narrowing of SC lumen that increases the shear stress on SC cells. Elevated shear stress stimulates NO production, which acts to reduce outflow resistance and relax trabecular meshwork cells to decrease trabecular meshwork stiffness, affecting the SC luminal caliber.

**Results:**

During constant flow perfusion, the outflow system is typically stable, returning to baseline IOP after a perturbation. In contrast, during constant pressure perfusion, the outflow system can become unstable and exhibit a time-dependent change in outflow resistance that diverges from baseline.

**Conclusions:**

The stability of shear mediated IOP homeostasis is predicted to differ critically between constant flow vs. constant pressure perfusion. Because outflow facility is typically measured at a constant pressure in the laboratory, this instability may contribute to the characteristic time-dependent increase in outflow facility, known as washout, observed in many nonhuman species. Studies of IOP homeostasis should consider how the outflow system may respond differently under constant pressure vs. constant flow perfusion.

Shear stress in Schlemm's canal (SC) is an important signal for IOP mechanosensation.^1^^–^^3^ In response to an IOP elevation, SC endothelial cells experience increased shear stress resulting from a pressure-induced narrowing of SC lumen.^4^ Elevated shear stress in turn stimulates nitric oxide (NO) production by SC cells,^5^^,^^6^ which are vascular in origin.^7^^,^^8^ Like vascular endothelial cells, SC cells express endothelial NO synthase^1^^,^^9^ that is responsible for shear-induced NO production. Exogenously delivered NO decreases outflow resistance,^9^^–^^13^ but more recent studies have revealed that endogenous NO produced in response to elevated SC shear stress^6^ modulates outflow resistance in situ.^14^^,^^15^ Shear-mediated NO production by SC cells is thus thought to provide a stabilizing feedback signal to oppose IOP perturbations,^2^ thereby contributing to IOP homeostasis and complimenting previously described mechanisms of stretch-induced IOP mechanosensation and homeostasis^16^ (Fig. 1).

**Figure 1.**
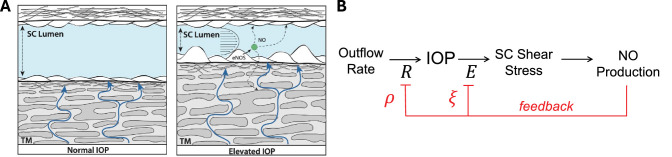
(**A**) Elevated IOP causes narrowing of SC lumen and increased shear stress acting on SC cells owing to circumferential flow of aqueous humor within SC. Shear stress stimulates NO production by endothelial NO synthase (eNOS) expressed by SC cells. (**B**) Shear-dependent NO production feeds-back to reduce both outflow resistance *R* and TM stiffness *E*. The sensitivities of *R* and *E* to NO are characterized by parameters *ρ* and *ξ*, respectively, per Equations 1 and 2. (A) Reproduced with permission from Reina-Torres et al., 2021^2^ © 2021 Elsevier Ltd.

An important factor in this homeostatic feedback pathway is the stiffness of the trabecular meshwork (TM) that tethers to the inner wall of SC and thereby controls the relationship between IOP and the caliber of SC lumen. As such, TM stiffness influences the shear stress experienced by SC cells and hence the quantity of NO production. However, this leads to the possibility of instability in the homeostatic IOP response. Namely, increased shear stress in SC increases NO production, which in turn relaxes TM cells^17^ and thereby decreases TM stiffness, allowing further SC collapse that further elevates shear stress in SC and further potentiates NO production. In this scenario, SC would progressively collapse and outflow resistance would progressively decrease, corresponding with a loss of IOP homeostasis.

We hypothesize that the balance between these opposing factors (stabilizing vs. destabilizing) depends on whether the outflow pathway is perfused under conditions of constant pressure vs. constant flow. Our rationale is as follows. Under physiological conditions, the outflow system is perfused at constant flow because the rates of aqueous humor secretion and unconventional outflow are relatively constant, changing slowly over the time scale of hours to days in human eyes^18^^,^^19^ and are largely pressure insensitive. When the inflow rate entering SC is constant, any NO-mediated decrease in outflow resistance and TM stiffness would tend to lower IOP, widen the SC lumen, and thus decrease the shear stress acting on SC cells. Conversely, most acute experimental ocular perfusions are conducted at constant pressure, dating back to the early works of Grant^20^ and Bárány.^21^ This is largely because constant pressure perfusion provides a faster measurement of outflow resistance relative to constant flow perfusion (see Supplemental Information 1), although constant flow is common for anterior segment organ culture perfusion over several days.^22^ Importantly, at constant pressure, the effect of NO on outflow resistance can no longer lower the IOP, which is fixed by the perfusion system. Instead, any increase in NO would increase the flow rate passing through the outflow pathway. This increased flow rate, combined with the effect of reduced TM stiffness owing to the increase in NO, would tend to further narrow SC and potentiate NO production, leading to the feed-forward (unstable) response described above.

To better understand these phenomena, we developed a mathematical model of shear-mediated NO production by SC endothelial cells, assuming an idealized SC anatomy and accounting for the effects of NO on outflow resistance and TM stiffness. Using a linearized model appropriate for small deviations around a baseline state, we explore the stability of the outflow system under conditions of constant pressure vs. constant flow perfusion. Stability is defined as the system being able to return to a baseline setpoint after a small perturbation, in contrast with an unstable system, which diverges from baseline after a small perturbation. Our study quantitatively predicts that the outflow system, which is normally stabilized by shear-mediated NO production under physiological (i.e., constant flow) conditions in vivo, may become unstable under conditions of constant pressure typically imposed during experimental ocular perfusion used to measure outflow facility.

## Methods

### Mathematical Formulation

We consider the flow of aqueous humor through the conventional outflow pathway, that is, through the TM and across the inner wall endothelium to enter the lumen of SC. Having entered SC, flow progresses circumferentially along the canal to exit via a collector channel ostium, from which aqueous humor returns to the systemic circulation. We simplify the mathematical formulation by proposing a zero-dimensional model, meaning that the model does not explicitly consider variations along the circumference of SC, but only variations in time. This approach is reasonable under conditions where there is low resistance to circumferential flow within SC lumen, as typically occurs at low to moderate IOPs,^3^ and where there is negligible anatomical variability around the circumference of the outflow pathway. All dimensional variables and parameters used in the formulation below are summarized in Supplemental Information 4.

Circumferential flow within SC generates a shear stress that stimulates NO production by SC endothelial cells. NO has two distinct roles. First, NO acts to decrease the combined hydrodynamic resistance of the inner wall and juxtacanalicular tissue (JCT), *R*, in proportion to the NO concentration within the lumen of SC, *C*, according to

(1) $$\begin{array}{c} R=R_{0}-\rho \left(C-C_{0}\right), \end{array}$$

where *R*_0_ is the reference value of *R* at the initial baseline condition, i.e. when the concentration of NO is equal to its baseline concentration *C*_0_. The parameter ρ is assumed to be positive and represents the decrease in flow resistance per unit increase in *C* above *C*_0_. Second, NO acts to induce relaxation and decrease TM stiffness, *E*, according to

(2) $$\begin{array}{c} E=E_{0}-\xi \left(C-C_{0}\right), \end{array}$$

where *E*_0_ is the reference value of *E* at baseline when *C* = *C*_0_. Like ρ, the parameter ξ is assumed to be positive and represents the decrease in *E* per unit increase in *C* above *C*_0_. Equations 1 and 2 capture the first-order effects of NO upon outflow resistance and TM stiffness, as appropriate when considering small perturbations about the baseline state.

The concentration of NO depends on its rate of production, which in turn depends on the shear stress τ acting on SC cells and the reactive decay of NO. The reactive decay of NO is attributable to reaction with heme-containing compounds or superoxide,^23^ described by first-order kinetics, and to a reaction with dissolved oxygen in aqueous solutions, described by second-order kinetics.^24^ Neglecting spatial variations in NO concentration along SC, the rate of change of *C* can thus be written as

(3) $$\begin{array}{c} \frac{dC}{dt}=\alpha \;\left(\tau -\tau_{0}\right)-\beta_{1}\;C-\beta_{2}\;C^{2}+\gamma , \end{array}$$

where γ is the baseline rate of NO production when τ is equal to the baseline value τ_0_, and α describes the increased rate of NO production per unit deviation in shear stress above τ_0_. Note that τ and τ_0_ are always positive because cells respond only to the magnitude of shear stress and because we expect no retrograde flow in SC under typical conditions.^3^ The nonlinear kinetics of Equation 3 allows one to explore the relative importance of first-order and second-order reactive decay of NO, which captures the reaction kinetics with heme or superoxide and oxygen, respectively, with corresponding reaction rate coefficients given by β_1_ and β_2_. Equation 3 predicts that the steady-state baseline value of *C* (when τ = τ_0_) is given by

(3b) $$\begin{array}{c} C_{0}=\frac{\sqrt{\beta_{1}^{2}+4\beta_{2}\gamma }-\beta_{1}}{2\;\beta_{2}}. \end{array}$$

Assuming laminar flow through SC lumen that has an approximately rectangular cross-section with an anterior–posterior width *w* that is much larger than the SC luminal interior–exterior height *h*, the shear stress acting on SC cells is given by

(4) $$\begin{array}{c} \tau =\frac{6\;\mu \;q}{w\;h^{2}}, \end{array}$$

where *q* is the local circumferential flow rate in SC (in the direction toward the collector channel ostium).^3^ Note that a positive value of τ acts in the flow-wise direction on both the inner and outer walls of SC.

Based on simulations by Sherwood et al.,^3^ the resistance to flow in SC at normal physiological IOP was less than 1% of the total outflow resistance in human eyes. If the baseline IOP lies within the normal physiological range, we may, therefore, neglect the resistance to flow through SC lumen. Under these conditions, the pressure becomes approximately uniform in SC, leading to a uniform value of *h* and a uniform filtration velocity across the inner wall. Consequently, both *q* and τ become linear functions of the flow-wise distance along SC, both taking a value of zero at the mid-point between collector channels and reaching a maximum at the collector channel ostium (cf. Fig. 4E and 4G of Sherwood et al.^3^). The values of *q* and τ considered in this zero-dimensional analysis thus represent spatial averages of these variables along the inner wall between collector channel ostia. As considered in greater detail in the Discussion, we note that these assumptions break down for high baseline IOP or low baseline SC height *h*_0_ or for abnormal canal geometries where the resistance to flow through the SC lumen becomes a significant fraction of total outflow resistance.

Following Sherwood et al.,^3^ *h* is related to the pressure drop across the inner wall Δ*P* according to

(5) $$\begin{array}{c} h=h_{r}\exp\left(-\frac{\Delta P}{E}\right), \end{array}$$

where *h_r_* is the resting height of SC in the absence of any applied pressure (Δ*P* = 0). This formulation accounts for the nonlinear stiffening of the TM with distension and prevents *h* from becoming negative, which could occur, for example, with a linear *h*-Δ*P* relationship. Note that *h_r_* ≠ *h*_0_, where *h*_0_ is the height of SC at baseline IOP. The pressure drop across the inner wall and TM, equivalent to the difference between IOP and the pressure in SC, is given by

(6) $$\begin{array}{c} \Delta P=Q\;R, \end{array}$$

where *Q* is the total flow rate through the conventional outflow pathway. *Q* is related to *q*, the spatially averaged flow rate in SC, according to *Q* = 4*Nq*, where *N* is the number of collector channel ostia. The factor of 4 arises because the flow rate near the collector channel ostium is twice the spatially averaged flow rate in SC (per the linear distribution in *q*, see above) and because the total flow through each ostium is doubled by the contribution from the portions of SC on either side of the ostium, which are assumed to be identical.

The value of IOP, represented by *P*, is given by Goldmann's equation

(7) $$\begin{array}{c} P=Q\;\left(R+R_{d}\right)+P_{e}, \end{array}$$

where *R_d_* is the value of distal outflow resistance and *P_e_* is the episcleral vessel pressure, both of which are assumed to be constant. Note that Equations 6 and 7 set a relationship between *P* and Δ*P*, which differ by a fixed amount (*Q*_0_ *R_d_* + *P_e_*) for constant flow perfusion, but vary independently of one another for constant pressure perfusion (owing to a varying *Q*). Unconventional outflow and aqueous inflow are not included in Equation 7 because only the conventional outflow *Q* is relevant for pressure regulation. The proximal outflow resistance *R* is NO sensitive, while the distal outflow resistance *R_d_* is assumed to be NO insensitive, despite recent data suggesting that NO may decrease distal resistance ex vivo.^25^ Because decreasing distal resistance promotes instability under constant pressure perfusion (see the Results), neglecting the effect of NO on distal resistance provides a conservative upper estimate on the stability threshold of IOP homeostasis.

Together, there are seven equations (Equations 1–7) defined for eight unknown variables (*C*, *Q* or *q*, *R*, *E*, τ, *h*, Δ*P*, and *P*). The final relationship necessary to complete the formulation is obtained from the defined state of the system, either as perfusion at constant pressure at *P* = *P*_0_ or constant flow perfusion at *Q* = *Q*_0_, where the subscript 0 refers to the baseline state. This dimensional formulation includes 15 independent parameters (*P*_0_ or *Q*_0_, ρ, *R*_0_, ξ, *E*_0_, α, β_1_, β_2_, γ, μ, *w*, *h_r_*, *N*, *R_d_*, and *P_e_*) from which it is convenient to define four additional dependent parameters that describe the baseline NO concentration *C*_0_ (per Equation 3b), the baseline height of SC:

(5b) $$\begin{array}{c} h_{0}=h_{r}\exp\left(-\frac{Q_{0}R_{0}}{E_{0}}\right), \end{array}$$

the baseline shear stress in SC:

(4b) $$\begin{array}{c} \tau_{0}=\frac{3\;\mu \;Q_{0}}{2\;w\;N\;h_{0}^{2}}, \end{array}$$

and the baseline value of IOP for constant flow perfusion at *Q* = *Q*_0_:

(7b) $$\begin{array}{c} P_{0}=Q_{0}\left(R_{0}+R_{d}\right)+P_{e}, \end{array}$$

or the baseline flow rate for constant pressure perfusion at *P* = *P*_0_:

(7c) $$\begin{array}{c} Q_{0}=\frac{P_{0}-P_{e}}{R_{0}+R_{d}}. \end{array}$$

By scaling the dimensional variables and reformulating in dimensionless terms, as described in the next section, we decrease the number of independent parameters from 15 to 6. This so-called nondimensionalization procedure is a standard technique used in fluid mechanics to decrease the complexity of a problem.

### Dimensionless Mathematical Formulation

We nondimensionalize the dependent variables by dividing each by their baseline value, and we indicate dimensionless quantities with superscript asterisks. For example, $C^{*}=\frac{C}{C_{0}}$ represents the dimensionless NO concentration, where *C* is normalized by the baseline NO concentration *C*_0_. We define the dimensionless time variable as $t^{*}=\frac{\gamma \;t}{C_{0}}$, where the characteristic time scale *C*_0_/γ represents the time needed to reach the baseline NO concentration at production rate γ. The full presentation of the dimensionless mathematical formulation is provided in Supplemental Information 2. This formulation results in eight dimensionless dependent variables and six dimensionless parameters that are summarized in the Table.

**Table.** Dimensionless Variables and Parameters


Dimensionless Variables	
$t^{*}=\frac{\gamma \;t}{C_{0}}$	Dimensionless time (independent variable)
$C^{*}=\frac{C}{C_{0}}$	Dimensionless NO concentration
$P^{*}=\frac{P}{P_{0}}$	Dimensionless IOP
$Q^{*}=\frac{Q}{Q_{0}}$	Dimensionless conventional outflow rate
$R^{*}=\frac{R}{R_{0}}$	Dimensionless outflow resistance of JCT & inner wall of SC
$E^{*}=\frac{E}{E_{0}}$	Dimensionless TM stiffness
$\Delta P^{*}=\frac{\Delta P}{\Delta P_{0}}$	Dimensionless pressure drop across JCT & inner wall of SC, $\Delta P_{0}=Q_{0}R_{0}$
$\tau^{*}=\frac{\tau }{\tau_{0}}$	Dimensionless shear stress in SC
$h^{*}=\frac{h}{h_{0}}$	Dimensionless height of SC
Dimensionless Parameters	
$\alpha^{*}=\frac{\alpha \;\tau_{0}}{\gamma }$	Ratio of shear-induced NO production rate (at τ = 2 τ_0_) to baseline NO production rate
$\eta^{*}=\frac{\beta_{2}\;C_{0}}{\beta_{1}}$	Relative importance of second-order to first-order reactive decay of NO
$\rho^{*}=\frac{\rho \;C_{0}}{R_{0}}$	Sensitivity of outflow resistance to NO, $\rho^{*}=-\frac{dR^{*}}{dC^{*}}$
$\xi^{*}=\frac{\xi \;C_{0}}{E_{0}}$	Sensitivity of TM stiffness to NO, $\xi^{*}=-\frac{dE^{*}}{dC^{*}}$
$h_{0}^{*}=\frac{h_{0}}{h_{r}}$	Ratio of baseline SC height to resting SC height, $h_{0}^{*}=\exp\left(-Q_{0}\;R_{0}/E_{0}\right)$
$R_{d}^{*}=\frac{R_{d}}{R_{0}+R_{d}}$	Ratio of distal to total outflow resistance at baseline

The resulting dimensionless governing equations (Equations 1–7) then become:

(1*) $$\begin{array}{c} R^{*}=1-\rho^{*}\left(C^{*}-1\right) \end{array}$$

(2a) $$\begin{array}{c} E^{*}=1-\xi^{*}\left(C^{*}-1\right) \end{array}$$

(3*) $$\begin{array}{c} \frac{dC^{*}}{dt^{*}}=\alpha^{*}\left(\tau^{*}-1\right)-\frac{1}{1+\eta^{*}}C^{*}-\frac{\eta^{*}}{1+\eta^{*}}{C^{*}}^{2}+1 \end{array}$$

(4*) $$\begin{array}{c} \tau^{*}=\frac{Q^{*}}{{h^{*}}^{2}} \end{array}$$

(5*) $$\begin{array}{c} h^{*}={h_{0}^{*}}^{\left(\frac{\Delta P^{*}}{E^{*}}-1\right)} \end{array}$$

(6*) $$\begin{array}{c} \Delta P^{*}=Q^{*}\;R^{*} \end{array}$$

(7*) $$\begin{array}{c} P^{*}=Q^{*}\;\left(R^{*}\left(1-R_{d}^{*}\right)+R_{d}^{*}\right). \end{array}$$

In the most general case, where both first- and second-order reactions are important, there are six dimensionless parameters in the system: $\alpha^{*},\;\eta^{*},\;\rho^{*},\;\xi^{*},\;R_{d}^{*}$, and $h_{0}^{*}$ (see the Table). Two of these parameters $\left(R_{d}^{*},\;h_{0}^{*}\right)$ can be defined based on knowledge of outflow anatomy or physiology. Two parameters (ρ*,  ξ*) describe the sensitivity of the outflow system to NO and are expected to be positive and no larger than approximately 2.0, which would represent a halving of *R* or *E* in response to a 25% increase in *C*, which we judge to be a reasonable upper limit of the physiological range. The dimensionless parameter α* = α_0_ τ_0_/γ describes the relative shear dependence of NO production by the inner wall, or more specifically the ratio of shear-induced NO production (α (τ − τ_0_)) after a doubling of shear stress above baseline (τ = 2 τ_0_) relative to the NO production rate at baseline (γ). A larger value of α* reflects a greater sensitivity of NO production to shear stress. The dimensionless parameter η* represents the relative importance of second-order vs. first-order NO decay kinetics. Note that, for the special cases when the system is either purely first-order (η* = 0) or purely second-order (η* → ∞), the contribution of η* vanishes in Equation 3*, leaving five dimensionless parameters.

### Stability Analysis

Equations 1* through 7* contain six algebraic relationships, but only one differential relationship. Thus, it is possible to express Equations 1* through 7* as a single nonlinear first-order ordinary differential equation for $\frac{dC^{*}}{dt^{*}}$. Following Strogatz,^26^ the stability of a such a one-dimensional system depends only on the slope of the relationship between $\frac{dC^{*}}{dt^{*}}$ and C*, given by $\frac{d}{dC^{*}}\left(\frac{dC^{*}}{dt^{*}}\right)$, evaluated at baseline (i.e., when all dimensionless variables take on the value of unity). Note that the baseline state is always in equilibrium because $\frac{dC^{*}}{dt^{*}}=0$ in Equation 3* whenever all dimensionless variables are equal to 1, but this baseline equilibrium may be stable or unstable. If $\frac{d}{dC^{*}}\left(\frac{dC^{*}}{dt^{*}}\right)\;>0$, then a perturbation that increases C* would correspond with a positive value of $\frac{dC^{*}}{dt^{*}}$, which in turn would further increase C*, and lead to a divergence from baseline. Such a response would indicate that the baseline condition is unstable. In contrast, if $\frac{d}{dC^{*}}\left(\frac{dC^{*}}{dt^{*}}\right)<0$, then a perturbation that increases C* yields a negative value of $\frac{dC^{*}}{dt^{*}}$ that opposes the perturbation and tends to return the system toward baseline, implying a stable equilibrium.

According to Equation 3*, the instability condition holds when$\;\alpha^{*}\frac{d\tau^{*}}{dC^{*}}>\frac{1+2\;\eta^{*}}{1+\eta^{*}}$ at baseline. The right-hand side of this inequality is equal to 1 for purely first-order reaction kinetics (when η* = 0) and is equal to 2 for purely second-order reaction kinetics (when η* = ∞). The left-hand side of the inequality can be expressed as $\frac{\alpha \tau_{0}}{\gamma }\frac{d\tau^{*}}{dC^{*}}$, which is a dimensionless ratio capturing the rate of NO production owing to increased shear stress in response to a perturbation vs. the NO production rate at baseline, γ. When this ratio exceeds 1 (for first-order) or 2 (for second-order), then the system becomes unstable. Physically, this means that the system becomes unstable when the production rate of NO in response to a perturbation roughly exceeds the baseline production rate.

The perturbation affects the rate of NO production by altering outflow resistance *R* and TM stiffness *E*, which together define the height of SC to influence shear stress. As such, the above inequality can be expressed in terms of the sensitivities of *E* and *R* upon *C*. Substituting for τ* in the inequality above predicts that the baseline state is unstable when

(8) $$\begin{array}{c} \frac{dQ^{*}}{dC^{*}}+2\left(\frac{dQ^{*}}{dC^{*}}+\xi^{*}-\rho^{*}\right)\ln\frac{1}{h_{0}^{*}}>\frac{1+2\;\eta^{*}}{\alpha^{*}\left(1+\eta^{*}\right)}, \end{array}$$

which holds either for constant flow or constant pressure perfusion. For the case of constant flow perfusion, $\frac{dQ^{*}}{dC^{*}}=0$, and Equation 8 predicts that baseline instability occurs when ξ* exceeds a critical value for constant flow $\xi_{c,Q}^{*}$ given by

(9) $$\begin{array}{c} \xi_{c,Q}^{*}=\rho^{*}+\frac{1+2\;\eta^{*}}{2\;\alpha^{*}\left(1+\eta^{*}\right)\ln\frac{1}{h_{0}^{*}}}. \end{array}$$

Physically, Equation 9 reveals that the system become unstable under constant flow when the sensitivity of TM stiffness to NO, ξ*, exceeds the sensitivity of outflow resistance to NO, ρ*, by more than a constant (i.e., the second term on the right-hand side of Equation 9). This constant depends inversely on α*, which defines the shear sensitivity of SC cells for NO production. With increasing shear sensitivity (increasing α*), the threshold for instability decreases because more NO is produced for any given value of shear stress, which drives the instability. Equation 9 sets a physiological upper limit for the value of ξ* because otherwise the in vivo state, which operates under constant flow, would not be able to maintain a stable IOP.

For the case of constant pressure perfusion, Equation 7* predicts that $\frac{dQ^{*}}{dC^{*}}=\left(1-R_{d}^{*}\right)\;\rho^{*}$ at baseline. Substituting this relationship into Equation 8 predicts that the baseline state is unstable for constant pressure perfusion when ξ* exceeds a critical value for constant pressure $\xi_{c,P}^{*}$ given by

(10) $$\begin{array}{c} \xi_{c,P}^{*}=\left(R_{d}^{*}+\frac{R_{d}^{*}-1}{2\;\ln\frac{1}{h_{0}^{*}}}\right)\rho^{*}+\frac{1+2\;\eta^{*}}{2\;\alpha^{*}\left(1+\eta^{*}\right)\ln\frac{1}{h_{0}^{*}}}. \end{array}$$

Comparing Equations 9 and 10 reveals that the two relationships differ only by the coefficient preceding ρ*. This coefficient depends on the contribution of distal outflow resistance and increases with increasing $R_{d}^{*}$, reaching unity when $R_{d}^{*}=1$. Thus, for all cases of physiological relevance where $R_{d}^{*}<1$, the coefficient preceding ρ* in Equation 10 is always less than unity, such that $\xi_{c,P}^{*}<\xi_{c,Q}^{*}$. Physically, this means that the threshold for instability under constant pressure perfusion is always less than that for constant flow perfusion.

### Physiological and Experimental Considerations

In the living eye, aqueous humor production and unconventional outflow are thought to be relatively constant, changing over hour-long time scales^18^^,^^19^ that are likely much slower than the temporal dynamics of NO within the outflow pathway (typically considered to occur over a time scale of seconds to minutes owing to the relatively fast turnover or decay of NO). Hence, under physiological conditions, the outflow system is effectively in a state of constant flow with the outflow rate equal to *Q*_0_, corresponding with Q* = 1. In the living eye, IOP is an output variable, determined by Goldmann's equation (Equations 7 or 7*).

Once the eye is connected to an external perfusion system to measure outflow facility, regardless of whether the perfusion is performed in vivo or ex vivo, the perfusion system can control either the flow rate or the pressure. We assume that the perfusion apparatus is ideal, in the sense that the perfusion apparatus can instantaneously provide any necessary flow or pressure to maintain the desired value of P* or Q*. Assuming an idealized perfusion apparatus operating at the baseline state allows us to isolate effects attributable the outflow system itself, while excluding external effects associated with filling times (as considered in Supplemental Information 1); environmental noise; imperfections in the perfusion apparatus, such as losses owing to perfusion resistance or compliance; or inaccuracies or delays in the perfusion control algorithm.

## Results

Equations 9 and 10 define the critical values of ξ* above which the outflow system becomes unstable during perfusion at constant flow ($\xi_{c,Q}^{*}$) or constant pressure ($\xi_{c,P}^{*}$), respectively. For any value of ξ* above the critical value, any perturbation however small would displace the system permanently away from baseline, indicating instability. Conversely, for any value of ξ* below the critical value, the system would be insensitive to small perturbations and would return to the baseline state, indicating stability. We now examine how the parameter values influence the stability of the outflow system, considering a particular case that approximates the human physiological state and a general case that applies to all parameter values.

### A Particular Case

We first consider a particular case where $R_{d}^{*}=0.25$, α* = 2.0, η* = 1.0, and $h_{0}^{*}=0.6$. These parameters correspond roughly to the human physiological state where distal resistance contributes one-quarter of total outflow resistance,^20^ where the SC lumen is 60% of its resting height at baseline, and where shear-induced NO production is roughly two-fold higher than baseline.^5^ The 60% resting height of SC is calculated, following Sherwood et al.,^3^ as $h_{0}^{*}=\exp\left(-Q_{0}\;R_{0}/E_{0}\right)$, where *Q*_0_ = 2.16 μl/min, *R*_0_ = 2.08 mmHg/(μl/min), and *E*_0_ = 8.2 mmHg, yielding $h_{0}^{*}=0.58\approx 0.6$. In the absence of literature data on NO reactivity in the human outflow pathway, we assume that first-order and second-order reaction kinetics are equivalent (hence η* = 1.0). We then allow ρ* and ξ* to vary over a presumed physiological range.

**Figure 2.**
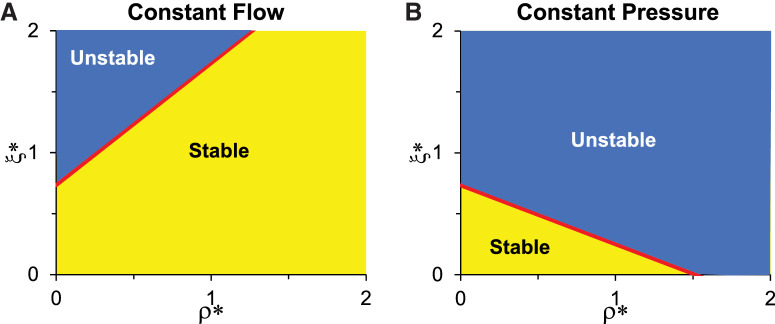
Domains of stability (*yellow*) and instability (*blue*) in the parameter space of ρ* and ξ* under constant flow (**A**) and constant pressure (**B**) perfusion. The red lines are the predictions of $\xi_{c,Q}^{*}$ and $\xi_{c,P}^{*}$ from Equations 9 and 10, respectively. The other dimensionless parameters are α* = 2.0, η* = 1.0, $R_{d}^{*}=0.25$ and $h_{0}^{*}=0.6$, corresponding approximately with the human physiological case.

Figure 2 shows the domains of stability and instability in the parameter space of ρ* and ξ* ranging from 0 to 2. Note that a value of ρ* or ξ* equal to 0 corresponds with NO having zero influence on *R* or *E*, whereas a value of 2 indicates that any increase in NO, say by an arbitrary amount given by *x*%, would have twice the effect on *R* or *E* (i.e. reducing *R* or *E* by 2*x*%). Such a range should reasonably encompass the physiological spectrum (from completely insensitive to NO to highly sensitive to NO).

For constant flow perfusion (Fig. 2A), the domain of instability (indicated in *blue*) is confined to the upper left of the parameter space, whereas most of the parameter space exhibits stability (indicated in *yellow*). The *red line* represents the critical value of $\xi_{c,Q}^{*}$ predicted by Equation 9, above which the baseline state becomes unstable. For constant pressure perfusion (Fig. 2B), the domain of instability (*blue region*) is larger than the domain of stability (*yellow region*). Further, the domain of instability for constant pressure perfusion is larger than that for constant flow perfusion (compare sizes of blue domains in Figures 2A and 2B). In other words, for all ρ^*^ > 0, the value of $\xi_{c,P}^{*}$ (indicated by the red line in Fig. 2B) is smaller than the corresponding value of $\xi_{c,Q}^{*}$ (*red line* in Fig. 2A). These results demonstrate that the baseline state is more prone to instability under constant pressure perfusion relative to constant flow perfusion, using parameters approximating the human outflow system. In the general analysis below, we show that for any selection of parameter values, the threshold for instability is always lower for constant pressure relative to constant flow perfusion (i.e., $\xi_{c,P}^{*}<\xi_{c,Q}^{*}$ for all cases of physiological relevance).

Numerical simulations of the time-dependent response of the outflow system to a small perturbation are consistent with the results shown in Figure 2. As described in Supplemental Information 3, a system with parameter values that lie within the stable domains of Figure 2 will, in response to a small perturbation, return to the baseline state. In contrast, a system with parameter values that lie within the unstable domains of Figure 2 will deviate permanently from baseline. For most points within the unstable domain, the deviation grows indefinitely (or until the system breaks down or saturates). Such large deviations from baseline are outside the scope of our formulated model and should be considered questionable. In the interest of brevity, we focus the remainder of the main text on the stability analysis and refer the interested reader to Supplemental Information 3 for further details on the detailed time-dependent response.

### General Analysis

**Figure 3.**
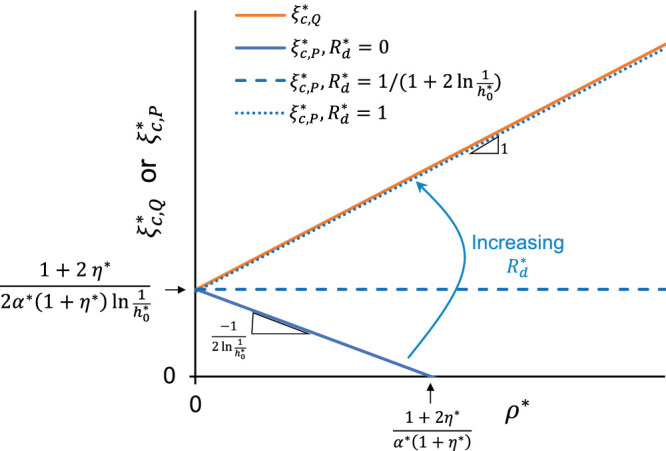
Diagram summarizing the general dependence of the critical values of ξ* on the dimensionless parameters ρ*, α*, η*, $h_{0}^{*}$, and $R_{d}^{*}$ for constant flow ($\xi_{c,Q}^{*}$, *orange line*; Equation 9) and constant pressure perfusion ($\xi_{c,P}^{*}$, *blue lines*; Equation 10). The unstable domain lies above either the *orange* or *blue lines*. Note that the slope of $\xi_{c,P}^{*}$ vs. ρ* increases with $R_{d}^{*}$, transitioning from the solid blue line when $R_{d}^{*}=0$ to the dotted blue line when $R_{d}^{*}=1$, where the slope for $\xi_{c,P}^{*}$ converges to that of $\xi_{c,Q}^{*}$. At an intermediate value of $R_{d}^{*}$, $\xi_{c,P}^{*}$ becomes independent of ρ* and exhibits a slope of zero (dashed blue line). In contrast, the slope of $\xi_{c,Q}^{*}$ vs. ρ* maintains a value of unity and is entirely insensitive to $R_{d}^{*}$.

To generalize how the instability threshold varies across the wider parameter space, we schematically map how $\xi_{c,Q}^{*}$ and $\xi_{c,P}^{*}$ depend on ρ* (Fig. 3). For constant flow perfusion (*orange tracing* in Fig. 3), $\xi_{c,Q}^{*}$ is represented as a linear function of ρ* with a slope of unity. The domain of instability lies above this line for all cases of constant flow perfusion. Note that the instability threshold for constant flow is independent of $R_{d}^{*}$. The intercept, representing the critical value of ξ* at ρ* = 0, is identical for both constant flow and constant pressure perfusion (compare the last terms in Equations 9 and 10), such that $\xi_{c,Q}^{*}=\xi_{c,P}^{*}$ at ρ* = 0. This intercept depends on α*, η*, and $h_{0}^{*}$. Increasing α* decreases the intercept, while increasing η* or $h_{0}^{*}$ increases the intercept.

For constant pressure (*blue tracings* in Fig. 3), $\xi_{c,P}^{*}$ has the same intercept as for $\xi_{c,Q}^{*}$, but the slope now depends on $R_{d}^{*}$. As in the constant flow case, the unstable region lies above the *blue lines*. If $R_{d}^{*}=0$ (*solid blue line* in Fig. 3), then $\xi_{c,P}^{*}$ decreases for increasing ρ* with a slope of −$1/(2\ln\frac{1}{h_{0}^{*}}),$ reaching $\xi_{c,P}^{*}=0$ at $\rho_{c,P}^{*}=\left(1+2\eta^{*}\right)/\left(\alpha^{*}\left(1+\eta^{*}\right)\right)$. Thus, zero distal resistance represents the most unstable case, with the lowest possible threshold for instability under constant pressure perfusion. Moreover, whenever $\rho^{*}\geq \rho_{c,P}^{*}$ for $R_{d}^{*}=0$ then, regardless of the value of ξ*, then all cases are unstable, even when the TM stiffness is entirely insensitive to NO.

As $R_{d}^{*}$ increases for constant pressure perfusion, holding $h_{0}^{*}$ constant, the slope increases until it reaches a value of unity when $R_{d}^{*}=1$ (*dotted blue line* in Fig. 3), at which point $\xi_{c,P}^{*}$ converges to $\xi_{c,Q}^{*}$. Thus, for any arbitrary set of parameter values, the instability condition for constant pressure perfusion lies between the two extremes set by $R_{d}^{*}=0$ and $R_{d}^{*}=1$. For the intermediate case when $R_{d}^{*}=1/\left(1+2\ln\frac{1}{h_{0}^{*}}\right)$ (*dashed blue line* in Fig. 3), $\xi_{c,P}^{*}$ becomes independent of ρ*, such that the slope is zero. These data reveal that increasing distal resistance promotes stability of the outflow system under constant pressure perfusion, yet $R_{d}^{*}$ has no effect on stability under constant flow perfusion.

To be consistent with our understanding of ocular physiology, the in vivo state must be stable at baseline; otherwise, the eye would not be able to maintain a constant IOP. Because the in vivo state is one of constant flow perfusion, the domain above the *orange line* in Figure 3 must thus be strictly prohibited. In other words, ξ* must always be less than $\xi_{c,Q}^{*}$, regardless of whether the outflow system is perfused at constant flow or constant pressure. This finding indicates that the parameter space that is consistent with physiology can be divided into two domains. The first “always stable” domain lies below the threshold for instability at constant pressure (i.e., $\xi^{*}<\xi_{c,P}^{*}$) and in this region the system is always stable, regardless of whether the eye is perfused at constant flow or constant pressure. The second “conditionally stable” domain lies above the threshold for instability at constant pressure, but below the threshold for instability under constant flow (i.e., $\xi_{c,P}^{*}<\xi^{*}<\xi_{c,Q}^{*}$), such that the response is stable under constant flow but unstable under constant pressure. We speculate that eyes from different species, or eyes from the same species under different physiological or pathological conditions, may occupy different locations within the available parameter space, possibly explaining why perfusion at constant pressure is stable in some species but unstable in others (e.g., the washout phenomenon; see the Discussion). The response depends on the parameter values appropriate for each species or physiological condition, where those parameter values lie within the domains of stability or conditional stability and, importantly, whether the eye is perfused under constant flow or constant pressure.

## Discussion

In this study, we proposed a mathematical model of NO-mediated IOP homeostasis, and we used this model to examine the stability of the baseline state of the conventional outflow pathway under conditions of constant flow vs. constant pressure perfusion. The model was motivated by recent studies showing that shear-induced NO production by SC cells may decrease outflow resistance and TM cell contractility, as a part of a fast-acting mechanism for IOP homeostasis.^1^^,^^2^^,^^14^ The baseline state is stable when, in response to a small perturbation, the system returns to the baseline setpoint, as one would expect from a functional homeostatic system that regulates IOP. In contrast, the baseline state is unstable when the system diverges from baseline, even in response to an infinitesimal perturbation, indicating a failure of IOP homeostasis. The model predicts that, for any given set of parameters, the threshold for instability is lower for perfusion at constant pressure relative to perfusion at constant flow, that is, there is greater tendency for instability when eyes are perfused at constant pressure vs. constant flow.

As the outflow system operates under constant flow in vivo, this means that any homeostatic response measured in an eye perfused at constant pressure may differ from the homeostatic response that may operate under constant flow. Because constant pressure perfusion provides a faster measurement of outflow resistance relative to constant flow (see Supplemental Information 1), most outflow measurements (especially for whole globes) are performed at constant pressure. Thus, the instability phenomenon described in this study may impact significantly the interpretation of IOP homeostasis based on experimental ocular perfusion performed under constant pressure.

### Mechanism of Instability

The mechanism for instability is attributable to the sensitivities of both proximal outflow resistance and TM stiffness to NO and the effect that these factors have on driving further changes in NO production by SC cells. For instance, if a small perturbation results in an increase in NO that, in turn, drives effects that further potentiate NO production, then the system would be unstable.

Consider the response to an infinitesimal perturbation in NO concentration under conditions of constant flow. In this case, the pressure drop Δ*P* across the inner wall and JCT is determined solely by the resistance *R* because the outflow *Q* is fixed. Therefore, a positive perturbation in NO would tend to decrease Δ*P* owing to the effect of NO on *R*. This phenomenon decreases the driving force for SC collapse, promoting an increase in SC height *h* and a decrease in shear stress τ on SC cells. In contrast, an increase in NO decreases TM stiffness *E*, which weakens the restoring force opposing SC collapse, promoting a decrease in *h* and an increase in τ. The balance between these two competing factors determines whether a perturbation in NO leads to an increase or decrease in τ that may further potentiate NO production, defining the conditions for instability under constant flow perfusion.

Presumably, under constant flow as exists in vivo, NO-mediated IOP homeostasis is operating within the stable domain of parameter values predicted by Equation 9, where any small perturbation would trigger a response that returns the outflow system toward the baseline setpoint. Otherwise, it would not be possible to maintain a stable IOP according to our model. However, when the eye is perfused at a constant pressure, as is typical when measuring outflow resistance, this limitation imposes a nonphysiological state that effectively “short-circuits” the shear-mediated homeostatic machinery, causing a divergence from a baseline setpoint that would otherwise be stable under constant flow.

To better understand how constant pressure perfusion may “short-circuit” shear-mediated IOP homeostasis, consider the following. An increase in NO would tend to reduce *R*, but because the pressure is fixed by perfusion, this decrease in *R* does not effectively decrease Δ*P*, as for the case of constant flow. Instead, an increasing perturbation in NO increases *Q*. At the same time, an increase in NO acts to decrease *E*, decreasing the restoring force opposing SC collapse, leading to a decrease in *h*. The combination of increasing *Q* and decreasing *h* acts synergistically to increase τ, which further promotes NO production. Contrast this synergy with the antagonistic response occurring under constant flow, where an increase in NO leads to competing effects on τ and NO production. This is the underlying reason for why perfusion at a constant pressure has a lower threshold for instability than perfusion at a constant flow.

### Effect of Distal Resistance on the Threshold for Instability

The model predicts that distal resistance acts to promote stability under constant pressure by increasing the threshold value for instability in Equation 10. In contrast, the stability threshold under constant flow is unaffected by distal resistance. To understand the role of *R_d_*, recognize that any increase in *Q* leads to a rise in the distal pressure drop. This in turn leads to a proportional decrease in Δ*P* if the total pressure drop is held constant. Thus, for constant pressure perfusion, an increase in *R_d_* drives a redistribution of the pressure drop from proximal to distal, which tends to decrease the driving force for SC collapse, which tends to decrease τ. In contrast, with a fixed *Q* under constant flow, Δ*P* is independent of distal pressure drop and, hence, *R_d_* has no effect on τ. This explains why increasing *R_d_* promotes stability under constant pressure perfusion but has no effect under constant flow perfusion.

### Effect of First- and Second-Order Decay of NO on the Threshold for Instability

Our analysis included the effects of both first-order and second-order decay of NO. First-order decay arises owing to reactions between NO and heme proteins^23^ present within the TM, sclera, or within the endothelial lining of SC; such proteins include hemoglobin, cytochromes, and soluble guanylyl cyclase, the latter of which is the principal mediator for the bioactive effects of NO on cell contractility. Alternatively, first-order decay may arise owing to reaction with superoxide.^23^ Second-order decay arises owing to a reaction with soluble oxygen, which has a stichometry of 2 moles of NO per mole of O_2._^24^ Assuming that the reactants other than NO are not rate limiting, any first- or second-order reactions can be grouped together into a single term with an appropriately modified reaction rate coefficient.

It is difficult to know which of these reactions (first- vs. second-order) may dominate in any given circumstance, and it is equally difficult to know the precise reaction rates. We, thus, formulated our model to include both reaction terms and normalized by the reaction rates, resulting in the dimensionless parameter η*, which captured the relative importance of second-order relative to first-order NO kinetics.

In our general analysis (Fig. 3), we found that changing η* influences $\xi_{c,Q}^{*}$ and $\xi_{c,P}^{*}$ only through the intercept at ρ* = 0, with η* having no effect on the slope in either case. In other words, a change in η* results in a uniform upward or downward shift in the relationship between the critical value of ξ* and ρ*. The magnitude of this shift is bounded between two limits. The lower limit corresponds with η* = 0, representing purely first-order decay, where the intercept is given by ${\left(2\alpha^{*}\ln\frac{1}{h_{0}^{*}}\right)}^{-1}$. The upper limit corresponds with η* → ∞, representing purely second-order decay, where the intercept is given by ${\left(\alpha^{*}\ln\frac{1}{h_{0}^{*}}\right)}^{-1}$. Thus, between the extremes of purely first-order vs. purely second-order decay, the intercept varies by a factor of 2. The second-order decay has a higher threshold, and is thus more stable, because second-order decay is faster than first-order at depleting NO, which is the component driving the instability. Nevertheless, this analysis reveals that, despite not knowing the precise reaction kinetics of NO within the outflow pathway, we can still put reasonable upper and lower limits on the critical thresholds for instability.

### Relationship to the Washout Phenomenon

Washout is a phenomenon in which outflow resistance progressively decreases during experimental ocular perfusion, which is typically performed at a constant pressure. Although the mechanism of washout remains unclear, understanding the basis for washout would provide insight into the mechanism of outflow resistance generation.^27^ For this reason, we developed our mathematical model to examine quantitatively the hypothesis that washout is caused by a “short-circuiting” of shear-mediated IOP homeostasis that manifests as an instability of the baseline state under constant pressure, but not constant flow, perfusion.

This hypothesis was experimentally tested by Kelly et al.,^15^ who demonstrated in porcine eyes perfused at constant pressure that the decrease in outflow resistance during washout is associated with increased nitrite levels in the perfusate, consistent with increased NO production. Further, perfusion with NO donors drove expansion of the JCT, as one would expect if NO caused TM/JCT relaxation.^15^ Likewise, enzymatic blockade of NO production with L-NAME suppressed washout and prevented JCT expansion.^15^ Thus, there is empirical evidence that runaway NO production in the outflow pathway contributes to washout in a manner consistent with predictions from our model.

Washout is observed in eyes from many species, including monkey,^21^^,^^28^^,^^29^ bovine,^30^^,^^31^ porcine,^32^ canine,^33^^,^^34^ feline,^34^ and rabbit.^35^ Although washout was originally attributed to a washing away of resistance generating material from the outflow pathway,^36^ more recent studies have attributed washout to a physical separation of the inner wall from the underlying JCT^30^^,^^31^^,^^37^ that disrupts the funneling effect involved in outflow resistance generation.^38^ Although our model did not explicitly account for inner wall–JCT connectivity, the model does account for factors controlling SC lumen height (Equation 5), which we expect to be related to JCT expansion as observed during washout.

Even though washout is observed in many species, it is curiously absent from human^28^^,^^31^ and mouse eyes.^39^ Some investigators have attributed the lack of washout to a well-developed cribriform plexus^40^^,^^41^ that tethers the inner wall endothelium to the JCT,^27^^,^^31^ and similar connections have been reported in mice.^42^ These connections, which presumably have a mechanical role to oppose SC collapse, would act to increase the effective TM stiffness and hence decrease the value of ξ*, which depends inversely on *E*. In this way, the cribriform plexus may promote the stability of outflow system to oppose washout by decreasing the value of ξ* below the critical values predicted by Equations 9 or 10.

Washout, however, has been observed in nonhuman primates in vivo^21^^,^^28^^,^^29^ which also have an extensive cribriform plexus.^41^ Thus, the presence or absence of the cribriform plexus on its own cannot fully explain the washout phenomenon. However, we point out that the critical threshold for instability depends on multiple factors given by Equations 9 or 10. For example, under constant pressure, any decrease in ξ* owing to the cribriform plexus may be offset by a decrease in $R_{d}^{*}$. Such differences in distal vessel resistance would presumably be related to interspecies differences in distal vessel architecture. Thus, in general, our model predicts that interspecies differences in washout could be explained by individual species occupying different locations in the available parameter space. Specifically, species that exhibit washout would lie in the “conditionally stable” domain, where $\xi_{c,P}^{*}<\xi^{*}<\xi_{c,Q}^{*}$, whereas species that do not exhibit washout would lie in the “always stable” domain, where $\xi^{*}<\xi_{c,P}^{*}$.

We recognize at least two inconsistencies in our model in relation to experimental observations of washout. The first is the prediction that, in response to a decreasing perturbation, the instability leads to a continuous increase in outflow resistance (see Fig. S3.2 of Supplemental Information 3), which is opposite the response observed in washout. To the best of our knowledge, we are not aware of any reports of inverse washout, where outflow resistance continually increases under constant pressure perfusion. However, there may be other mechanisms not considered in our model that prevent a runaway increase in outflow resistance, because this would otherwise lead to unchecked hypertension. Indeed, there are likely additional feedback mechanisms,^16^^,^^43^^,^^44^ not considered here, that help to maintain IOP homeostasis, and it would be of interest in future work to consider the interactions between the NO-mediated homeostatic system and these other feedback loops. Second, although we assume that the physiological state of constant flow should always be stable, this supposition contrasts with a limited number of studies that have reported washout under constant flow or had allowed washout to progress before reaching a stable outflow facility.^25^^,^^45^^–^^49^ Although we cannot explain this discrepancy, a possible explanation is that if the eyes were initially pressurized using a constant-pressure approach (e.g., to rapidly fill the eye to an anticipated IOP), even briefly, then this initial period of constant pressure could have initiated an instability that persists when the perfusion is reverted to constant flow. Alternatively, the loss of ciliary muscle tone after enucleation could decrease the effective TM stiffness, decreasing *E* and hence increasing ξ*, which could result in washout if the threshold given by Equation 9 were crossed. Time-dependent loss of distal vessel tone could potentially contribute to washout as well,^25^ as could conditions during enucleation or treatment of the animals before death.

### Limitations

Our model assumes a linear relationship between NO concentration and outflow resistance and TM stiffness, as given by Equations 1 and 2. Likewise, we assumed a linear relationship between shear stress in SC and the rate of NO production (Equation 3). The true relationships approximated by these equations are almost certainly nonlinear. However, in the context of a stability analysis, any nonlinear function can be linearized about an arbitrary state, as long as the deviations from that state remain small. For this reason, our analysis is limited to the baseline state and small perturbations about the baseline. The analysis predicts whether perturbations decay or grow, indicating stability or instability, respectively. For similar reasons, our model is unable to reliably predict the existence of additional stable states removed from the baseline state, because nonlinearities or saturation may become important at such nonbaseline states.

The model neglects spatial variations of all dimensionless variables within SC, by which we mean that we have assumed that all dimensionless variables do not depend on circumferential location. Evaluating the validity of this assumption is complex; therefore, we consider its validity for different aspects of the model, beginning with the assumption of spatially uniform SC caliber, *h*. This assumption is reasonable when two conditions are met. First, there must be little resistance to circumferential flow within SC. More specifically, the pressure drop owing to circumferential flow within the lumen should be small compared with the pressure drop across the TM/inner wall of SC, Δ*P*. This assumption will be violated when SC is highly collapsed, or when IOP is highly elevated,^3^^,^^50^ or where herniations of SC protrude into the collector channel ostia.^51^ Thus, our analysis is limited to normotensive conditions and small perturbations around the baseline state. Second, the hydrodynamic flow resistance of the TM/inner wall, *R*, and TM stiffness, *E*, must be spatially uniform. Because both of these quantities depend on NO concentration, this essentially requires that circumferential variations in *C* be small, a point that we return to below.

We now consider the flow rate within the lumen of SC. Even if Δ*P* and *h* are spatially uniform, there is definite spatial nonuniformity in *q*, and hence in τ. This follows trivially from the conservation of mass applied to aqueous humor. As fluid filters across the inner wall, *q* (and hence τ) must increase within SC. However, in our nondimensionalization we normalize τ by its local baseline value, τ_0_, which also varies spatially. By limiting our analysis to small perturbations about the baseline, the spatial variation in τ is the same as that for τ_0_, and thus the quotient τ* is essentially independent of location. This emphasizes the usefulness of using nondimensional variables in the stability analysis.

The final, and most complex, assumption is that there is a spatially uniform NO concentration in the lumen of SC. Based on the spatial variations in τ, and the shear dependence of NO production, there must be spatial variation in the local NO production rate. However, at steady state, the local NO concentration in the lumen of SC depends not only on the production rate of NO, but also on advective and diffusional mass transport along the canal and into the adjacent sclera and TM, where NO may undergo reactive decay. An order of magnitude analysis suggests that, in both human and mouse eyes, the rate of circumferential mass transport is sufficiently rapid that NO is fairly well-mixed within SC, consistent with the assumption of a spatially uniform luminal concentration of NO. A more thorough analysis that properly accounts for spatial variations should be undertaken, because it would provide further insight into the distribution of NO within the outflow pathway and how the NO concentration may affect IOP homeostasis.

Finally, our model also does not consider the effects of NO on distal resistance, despite recent data showing that exogenous NO decreases distal resistance in both human and porcine anterior segments after contraction with endothelin-1.^25^ Under constant flow, our analysis suggests that $R_{d}^{*}$ has no effect on the stability threshold, and hence any effect of NO on distal resistance is likely unimportant for constant flow perfusion. Under constant pressure, however, $R_{d}^{*}$ influences the stability threshold, increasing $\xi_{c,P}^{*}$ as $R_{d}^{*}$ approaches unity. Because we expect NO to lower $R_{d}^{*}$, and hence decrease $\xi_{c,P}^{*}$, then any effect of NO on distal resistance should promote instability under constant pressure perfusion. Estimates of $\xi_{c,P}^{*}$ given by Equation 10 thus represents an upper limit, which may be decreased by the action of NO on distal resistance.

## Summary and Conclusions

We developed a mathematical model that captures the primary factors regulating shear-mediated IOP homeostasis via NO signaling in the aqueous humor outflow pathway. We then used this model to investigate the stability of the outflow system in response to small perturbations under conditions of either constant flow or constant pressure perfusion. The model predicts that the baseline state may become unstable owing to hyperactive feedback mechanisms that push the system away from equilibrium. Importantly, the critical threshold for instability is lower under conditions of constant pressure perfusion vs. constant flow perfusion, implying that experimental conditions imposed to measure outflow facility may affect any putative homeostatic mechanism. Moreover, this instability may contribute to the washout phenomenon, which is typically observed during constant pressure perfusion, and may explain why washout is observed in some species but not others. This model provides a quantitative framework for future attempts to understand IOP homeostasis and to guide targeted strategies that aim to decrease outflow resistance and IOP for glaucoma therapy.

## Supplementary Material

Supplement 1

Supplement 2

Supplement 3

Supplement 4
