# Supplementary material for: The Factors Affecting the Stability of IOP Homeostasis
Source: Invest Ophthalmol Vis Sci. 2024 Jun 4;65(6):4. doi: 10.1167/iovs.65.6.4 (PMC11157970; doi:10.1167/iovs.65.6.4)
Supplement: Supplement 1 [file iovs-65-6-4_s001.pdf]

A Mathematical Model of the Time-Varying Intraocular Pressure during Constant Pressure versus Constant Flow Perfusion

Supplemental Information 1 to

The Factors Affecting the Stability of IOP Homeostasis

Darryl R. Overby, C. Ross Ethier, Changxu Miao, Ruth A. Kelly, Ester Reina-Torres, W. Daniel Stamer

This supplement formulates a mathematical model of the time-varying intraocular pressure in response to step-change during either constant flow or constant pressure perfusion. This analysis assumes a passive outflow system with constant outflow resistance, independent of any homeostatic mechanisms, such as those considered in the main text. The goal is to estimate and compare the time required to reach equilibrium during perfusion of a passive (i.e., non-homeostatic) eye under conditions of constant flow versus constant pressure perfusion.

Formulation

Pressure-Volume Relationship of the Eye

We assume that the eye behaves as an elastic corneoscleral shell with a pressure-volume relationship given by Friedenwald's equation[1]:

$$\frac{dP}{P} = k\frac{dV}{V} \qquad \text{Eq. S1.1}$$

where P and V are the intraocular pressure and ocular volume, respectively, and k is a dimensionless constant. As the ocular volume changes little relative to the reference state, i.e. the state where $V = V_0$, then the ratio k/V is nearly constant and approximately equal to

$$K = \frac{k}{V_0} \qquad \text{Eq. S1.2}$$

where K is the coefficient of ocular rigidity. A typical value of K for human eyes is $0.025\ \mu l^{-1}$ [ref. 1].

Integrating Equation S1.1 using the definition for K yields

$$P = P_0 \exp\left[K(V - V_0)\right] \qquad \text{Eq. S1.3}$$

Where we have applied the condition that $P = P_0$ at $V = V_0$, i.e. at the reference state. Equation S1.3 prescribes the pressure volume relationship of the eye accounting for pressure-induced stiffening of the corneoscleral shell, as predicted by Friedenwald's relationship. Differentiating Equation S1.3 yields

$$\frac{dP}{dt} = K\,P\frac{dV}{dt} \qquad \text{Eq. S1.4}$$

which is a relationship needed to complete the time-dependent formulation below. Equation S1.4 could also be obtained directly from Equations S1.1 and S1.2.

Conservation of Mass Applied to Aqueous Humor or Perfusate

We assume that aqueous humor or perfusate is an incompressible fluid that occupies some portion of V within the eye. We assume that the remaining portion of V (e.g., vitreous humour, lens, retina) has constant volume, such that any change in V is proportional to the change in volume of aqueous humor or perfusate within the eye. The conservation of mass can then be written as

$$\frac{dV}{dt} = Q_{in} - Q_{out} \qquad \text{Eq. S1.5}$$

where Q_{in} is the inflow of fluid into the eye, while Q_{out} is the total outflow. In general, Q_{in} may have contributions from the natural production of aqueous humour, Q_n, as well as inflow from the perfusion system, Q_p, such that

$$Q_{in} = Q_n + Q_p \qquad \text{Eq. S1.6}$$

While Q_n is assumed to be a constant, Q_p depends on whether the perfusion is performed under conditions of constant flow or constant pressure, as described below.

We consider a combination of pressure-dependent outflow plus pressure-independent outflow, Q_u, the latter of which we assume constant. We can thus write the total outflow Q_{out} as

$$Q_{out} = \frac{P - P_e}{R} + Q_u \qquad \text{Eq. S1.7}$$

where R is the hydraulic resistance of pressure-dependent outflow and P_e is the episcleral venous pressure, where both R and P_e are assumed to be constants. For enucleated or post-mortem eyes, $Q_n = 0$ and $P_e = 0$.

Generalized Governing Equation

We can combine Equations S1.4-7 to obtain

$$\frac{dP}{dt} = K\,P\left(Q_n + Q_p - Q_u + \frac{P_e}{R} - \frac{P}{R}\right) \qquad \text{Eq. S1.8}$$

which is a non-linear ODE that describes the time-dependent pressure response of the eye $P(t)$ during constant flow or constant pressure perfusion. To solve Equation S1.8

requires a formulation for Q_p, but this depends on whether the perfusion is performed under constant flow or constant pressure.

For constant flow perfusion, the eye is typically connected to a pump that controls Q_p, with the flow entering the eye through a cannula threaded through the cornea. We assume an ideal constant flow perfusion system, which necessitates zero system compliance upstream of the eye (i.e., perfectly rigid tubing). Under this assumption, the volume flow rate into the eye Q_p is exactly equal to the output from the pump. Equation S1.8, with Q_p being a constant, thus describes constant flow perfusion.

In the case of constant pressure perfusion, the eye is typically connected to a pressure source, such as a fixed height reservoir, with the flow entering the eye through a cannula. If the pressure source maintains a constant applied pressure P_a upstream of the cannula, then the flow rate entering the eye varies, depending on the pressure drop across the cannula, such that

$$Q_p = \frac{P_a - P}{R_c} \qquad \text{Eq. S1.9}$$

where R_c is the hydraulic resistance of the cannula. This assumes negligible resistance elsewhere in the perfusion system upstream of the cannula. Thus, the governing equation for constant pressure perfusion becomes

$$\frac{dP}{dt} = K\,P\left(Q_n + \frac{P_a}{R_c} - Q_u + \frac{P_e}{R} - P\left(\frac{1}{R} + \frac{1}{R_c}\right)\right) \qquad \text{Eq. S1.10}$$

Note that Equations S1.8 and S1.10 have the same form, meaning that both can be written as

$$\frac{dP}{dt} = K\,P\left(Q_+ - \frac{P}{R_+}\right) \qquad \text{Eq. S1.11}$$

where Q_+ and R_+ are constants representing an effective pressure-dependent outflow rate and outflow resistance defined as

$$Q_+ = \begin{cases} Q_n + Q_p - Q_u + \dfrac{P_e}{R} & for\ constant\ flow \\ Q_n + \dfrac{P_a}{R_c} - Q_u + \dfrac{P_e}{R} & for\ constant\ pressure \end{cases} \qquad \text{Eq. S1.12}$$

and

$$R_+ = \begin{cases} R & for\ constant\ flow \\ \dfrac{R\,R_c}{R + R_c} & for\ constant\ pressure \end{cases} \qquad \text{Eq. S1.13}$$

The steady state solution to Equation S1.11 reduces to a form that is consistent with Goldmann's equation and is given by

$$P_\infty = R_+\,Q_+ \qquad \text{Eq. S1.14}$$

where P_∞ is the steady state pressure drop across the pressure-dependent outflow pathway.

Equation S1.14 allows one to measure outflow resistance R at a controlled pressure or flow rate once pressure equilibration is reached. This requires the ability to measure the perfusion flow rate into the eye at the applied pressure P_a or to measure P_∞ at the prescribed value of Q_p, as well as knowing any additional necessary parameters R_c, Q_n, Q_u, and P_e. This process is often repeated over multiple steps of pressure or flow, for example to determine whether R is pressure dependent. However, in all instances the perfusion must reach pressure equilibration to obtain an accurate measurement of R at each step. The question then becomes: how much time is required to reach equilibration, and does this time differ between constant pressure and constant flow perfusion?

General Solution

Equation S1.11 can be solved by separation of variables with integration by partial fractions. Applying the initial condition that $P = P_0$ at $t = 0$ yields the general solution

$$P = \frac{P_\infty}{1 + \left(\frac{P_\infty}{P_0} - 1\right) \exp\left[-\frac{t}{\tau}\right]} \qquad \text{Eq. S1.15}$$

where the time constant τ is defined as

$$\tau = \frac{R_+}{K\, P_\infty} \qquad \text{Eq. S1.16}$$

Note that τ varies between constant pressure and constant flow perfusion due to the dependence of R_+ on perfusion conditions, per Equation S1.13.

It is useful to non-dimensionalize Equation S1.15 to obtain

$$P^* = \frac{1}{1 + \left(\frac{1}{P_0^*} - 1\right) \exp[-t^*]} \qquad \text{Eq. S1.17}$$

where $P^* = P/P_\infty$, $t^* = t/\tau$, and $P_0^* = P_0/P_\infty$. Note that P_0^* represents the dimensionless value of the initial pressure. The form of Equation S1.17 is shown in Figure S1.1 for different values of P_0^*, where the non-linearity manifests as a change in the shape of the curves across the different values of P_0^*. Note also that the response time appears faster for $P_0^* > 1$ and slower for $P_0^* < 1$.

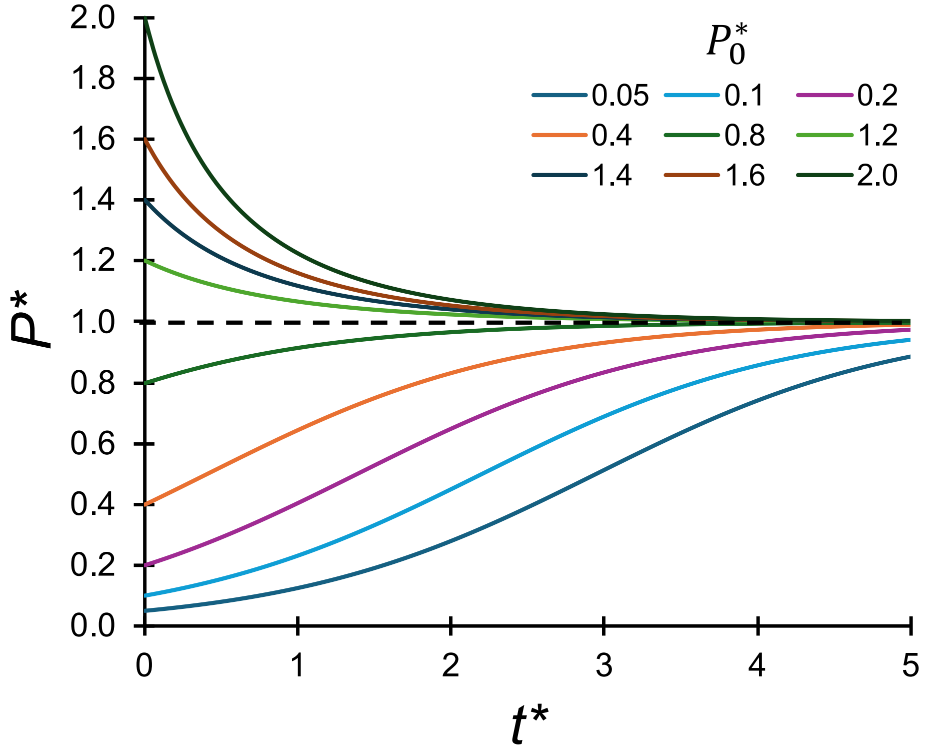

Figure S1.1: A plot of Equation S1.17 for different values of the dimensionless starting pressure P_0^*. The plot holds for both constant pressure and constant flow perfusion, but note that the time constant τ, which sets the time scale, differs between the two cases, per Equations S1.13 and S1.16.

Time to Reach Equilibrium

The solution given by Equation S1.15 asymptotically approaches P_∞ as $t \to \infty$. Thus, to calculate the time to reach equilibrium, we define an approximate equilibrium when P reaches some quantity P_ε that is arbitrarily close to P_∞. We define this quantity based on the following ratio:

$$\varepsilon = \frac{P_\infty - P_\varepsilon}{P_\infty - P_0} = \frac{1 - P_\varepsilon^*}{1 - P_0^*} \qquad \text{Eq. S1.18}$$

where $P_\varepsilon^* = P_\varepsilon / P_\infty$ is the dimensionless value taken by P^* when the system has reached approximate equilibrium and where ε is a constant often taken to be 5%.

The time to reach approximate equilibrium, t_ε, is thus the time when P^* reaches $P_\varepsilon^* = 1 - \varepsilon(1 - P_0^*)$. Solving for t_ε yields

$$t_\varepsilon = \tau \left(\ln\left[\frac{1}{\varepsilon}\right] + \ln\left[\frac{1 - \varepsilon(1 - P_o^*)}{P_o^*}\right] \right) \qquad \text{Eq. S1.19}$$

Note that for a linear response given by $P^* = 1 - (1 - P_0^*)\exp[-t/\tau]$, which differs from the solution above, then t_ε would be given by $\tau \ln[1/\varepsilon]$, which is the first term in Equation S1.19 and yields the typical $t_\varepsilon \approx 3\,\tau$ for $\varepsilon = 0.05$. Thus, the non-linear response of the eye given by Equation S1.15 causes the time to reach equilibrium to deviate from the typical linear estimate by an amount that depends on P_o^*. Note that when $P_o^* = 1$, the second term, which can also be written as $\ln[P_\varepsilon^*/P_o^*]$, vanishes from Equation S1.19, leaving only the linear estimate for t_ε.

Figure S1.2 shows how the predicted values of t_ε (normalized by τ) depend on P_0^* for different values of ε. Note that Equation S1.19 reproduces the typical linear case of "$3\,\tau$" when $\varepsilon = 0.05$ and $P_0^* = 1$, and as expected, reducing ε requires longer to reach approximate equilibrium because P_ε is set nearer to P_∞. However, Equation S1.19 also predicts that t_ε increases with decreasing P_0^*. This means that the time to reach equilibrium is longer for smaller values of P_0^*, as also can be appreciated from the tracings in Figure S1.1. For $P_0^* < 1$, the time response is slower than the linear case (represented by the intercept at $P_0^* = 1$), while for $P_0^* > 1$, the time response is faster than the linear case. The origin of this asymmetry arises from the pressure-induced stiffening of the corneoscleral shell, as predicted by Friedenwald's equation, with the increased stiffness driving a faster temporal response at higher pressures. Equation S1.19 provides real-world benefit by showing that perfusion protocols that step-down in pressure or flow (i.e., that start from an initial pressure that is larger than the equilibrium pressure) will achieve a faster equilibrium response than perfusion protocols that step-up in pressure or flow (i.e., that start from an initial pressure that is lower than equilibrium).

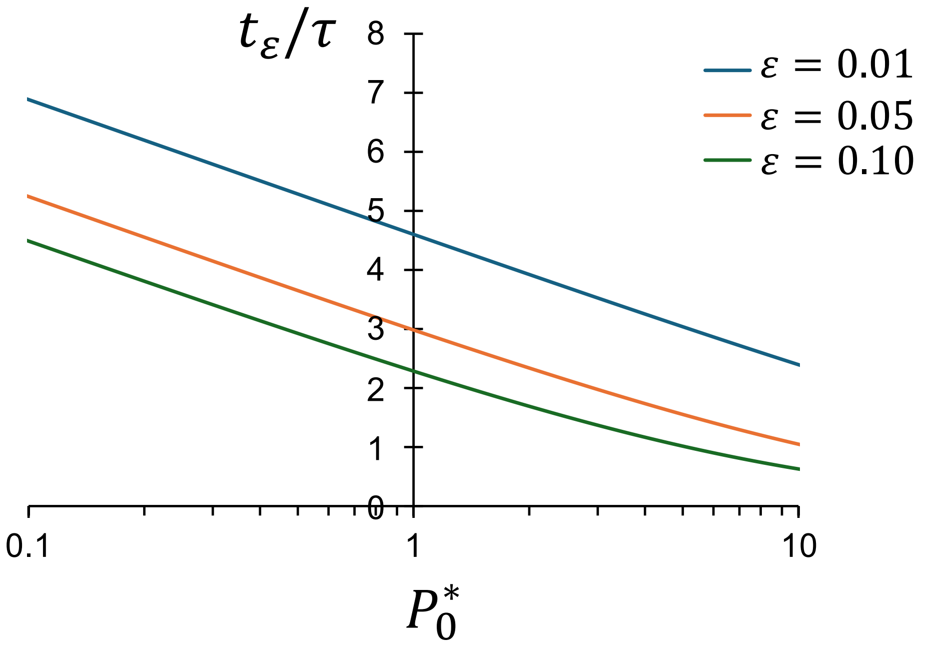

Figure S1.2: The time to reach approximate equilibrium t_ε, per Eq. S1.19, normalised by the time constant τ. This relationship holds for both constant pressure and constant flow perfusion, with the proviso that τ differs between the two cases according to Eqs. S1.13 & S1.16. Note that for $\varepsilon = 0.05$ at $P_0^* = 1$, then $t_\varepsilon \approx 3\tau$, as expected for a typical linear response. Note the log scale of P_0^*.

Comparing Time Constants Between Constant Pressure and Constant Flow Perfusion

Equation S1.15 is a general solution that is valid for perfusion at either constant pressure or at constant flow. Thus, the form of the time-dependent response is identical between the two cases. However, the time constant τ differs according to

$$\tau = \begin{cases} \tau_q = \dfrac{R}{K\,P_\infty} & for\ constant\ flow \\ \tau_p = \dfrac{R\,R_c}{K\,P_\infty\,(R+R_c)} & for\ constant\ pressure \end{cases} \qquad \text{Eq. S1.20}$$

Note that, since we expect $R_c \ll R$, then τ_p is approximately $R_c/K\,P_\infty$, which depends on R_c but not R, while τ_p always depends on R.

We now consider two cases where an otherwise identical eye is perfused at constant pressure versus constant flow. More specifically, we consider the transient response of the eye for a step change in pressure or flow, respectively. If for the two cases, the initial and final pressures P_0 and P_∞ are the same, then the time to reach the same value of any intermediate pressure is always longer for the case of constant flow relative to constant pressure perfusion. The ratio of the times required to reach any intermediate pressure is equal to the ratio of the time scales and is given by

$$\frac{\tau_q}{\tau_p} = 1 + \frac{R}{R_c} \qquad \text{Eq. S1.21}$$

As for all cases of practical interest $R \gg R_c$, then we expect $\tau_q \gg \tau_p$. Equation S1.21 can be obtained directly from Equation S1.19 by simply taking the ratio of the two conditions for t_ε, holding P_0^* and ε fixed.

Equation S1.21 demonstrates that, all else being equal, the time to reach steady state (or any intermediate state) is always longer under constant flow perfusion relative to constant pressure perfusion and could typically be much longer. This occurs because when the resistance of the cannula is small, τ_p is approximately equal to $R_c/K\,P_\infty$, which is independent of R, while τ_q remains unaffected by low cannula resistance and depends on the value of total outflow resistance R. In other words, reducing R_c will reduce τ_p, but will not reduce τ_q. Stated slightly differently, a constant pressure perfusion more rapidly accommodates the volumetric change of the eye that must occur in response to the step change relative to constant flow perfusion.

To provide quantitative estimates for the time to reach equilibrium, we apply the parameter values shown in Table S1, which approximate human eyes. We calculate τ and the typical estimate of 3τ, which assumes the ideal case where P_0 starts very near P_∞ with $\varepsilon = 0.05$. Note that if P_0 differs from P_∞ or if ε differs from 0.05, then the estimate for t_ε would need to be revised per Equation S1.19 or as shown in Figure S1.2. These estimates show that the time to reach approximate equilibrium at constant flow perfusion can be 6- to 280-fold longer than that for constant pressure perfusion. For this reason, most ocular perfusion measurements of enucleated eyes are conducted at constant pressure, rather than constant flow, perfusion.

Table S.1: Estimates of time to reach equilibrium based on Equation S1.19, assuming $P_0^* = 1$ (corresponding to the linear case) and $\varepsilon = 0.05$.

Parameter	Value	Reference
K	0.025 μl^{-1}	ref. 1
P_∞	15 mmHg	Normotensive IOP
R	2.78 mmHg/(μl/min)	ref. 2
R_c	0.01-0.5 mmHg/(μl/min)	ref. 3
$\frac{\tau_q}{\tau_p} = 1 + \frac{R}{R_c}$	6.6 - 280	Equation S1.21
	**Constant Flow**	**Constant Pressure**
R_+	2.78 mmHg/(μl/min)	0.01-0.42 mmHg/(μl/min)
τ	7.4 min	0.03 – 1.1 min
$t_\varepsilon = 3\tau$	22.2 min	0.1 - 3.4 min

References

1. Gloster, J. *Tonometry and Tonography*. (J&A Churchill LTD, 1966).
2. Sherwood, J. M., Stamer, W. D. & Overby, D. R. A model of the oscillatory mechanical forces in the conventional outflow pathway. *J. R. Soc. Interface* **16**, 20180652 (2019).
3. Sherwood, J. M. *et al.* Measurement of ocular compliance using iPerfusion. *Front. Bioeng. Biotechnol.* **7**, (2019).
