# Supplementary material for: The Factors Affecting the Stability of IOP Homeostasis
Source: Invest Ophthalmol Vis Sci. 2024 Jun 4;65(6):4. doi: 10.1167/iovs.65.6.4 (PMC11157970; doi:10.1167/iovs.65.6.4)
Supplement: Supplement 2 [file iovs-65-6-4_s002.pdf]

Dimensionless Mathematical Formulation

Supplemental Information 2 to
The Factors Affecting the Stability of IOP Homeostasis

Darryl R. Overby[1], C. Ross Ethier[2], Changxu Miao[1], Ruth A. Kelly[3], Ester Reina-Torres[1], W. Daniel Stamer[3]

This supplement provides the detailed formulation of the dimensionless governing equations and parameters that appear in Equations 1*-7* and in Table 1 of the main text.

We define the dimensionless nitric oxide (NO) concentration as $C^* = C/C_0$, representing the relative change in C with respect to its initial baseline value C_0. Equation 3 from the main text can then be written as

$$\frac{dC^*}{dt^*} = \alpha^*(\tau^* - 1) - \frac{1}{1+\eta^*}C^* - \frac{\eta^*}{1+\eta^*}C^{*2} + 1 \qquad \text{Eq. S2.1}$$

where we have defined the dimensionless time variable as $t^* = \gamma\, t/C_0$, and we have introduced the dimensionless parameters $\alpha^* = \alpha\, \tau_0/\gamma$, which represents the ratio of shear-induced NO production to baseline NO production, and $\eta^* = \beta_2\, C_0/\beta_1$, where η^* represents the relative importance of second-order to first-order decay at the reference NO concentration. In obtaining the above relationship, it is useful to recognize that $\gamma/\beta_1 C_0 = 1 + \eta^*$, which may

be obtained from Equation 3 of the main text applied at steady state. The dimensionless shear stress $\tau^* = \tau/\tau_0$ is defined with respect to the baseline shear stress τ_0.

Defining the dimensionless hydraulic resistance of the inner wall and juxtacanalicular tissue (JCT) as $R^* = R/R_0$, Equation 1 from the main text becomes

$$R^* = 1 - \rho^*(C^* - 1) \qquad \text{Eq. S2.2}$$

where $\rho^* = \rho\, C_0/R_0$ represents the relative decrease in R^* per unit relative increase in C^* such that $\rho^* = -dR^*/dC^*$ or, alternatively $\rho^* = (1 - R^*)/(C^* - 1)$. This latter expression provides a meaningful interpretation for ρ^*. For example, a value of ρ^* equal to 0.1 means that R^* decreases by 10% in response to a doubling of C^*. Likewise, defining the dimensionless trabecular meshwork (TM) stiffness as $E^* = E/E_0$ reduces Equation 2 from the main text to

$$E^* = 1 - \xi^*(C^* - 1) \qquad \text{Eq. S2.3}$$

where $\xi^* = \xi\, C_0/E_0$ represents the relative decrease in E^* per unit relative increase in C^* or $\xi^* = (1 - E^*)/(C^* - 1)$, which has a similar interpretation to ρ^* above.

As q and Q are linearly related by the number of collector channels, $q = Q/4N$, any relative change in q is equal to the relative change in Q. We may express the dimensionless shear stress in Schlemm's canal (SC; Equation 4 of the main text) as

$$\tau^* = \frac{Q^*}{{h^*}^2} \qquad \text{Eq. S2.4}$$

where $Q^* = Q/Q_0$ and $h^* = h/h_0$ represent the dimensionless flow rate through the entire conventional outflow pathway and the dimensionless SC height, relative to the baseline values Q_0 and h_0. At the baseline state, $\Delta P_0 = Q_0\, R_0$, such that Equation 6 from the main text can be re-written as:

$$\Delta P^* = Q^*\, R^* \qquad \text{Eq. S2.5}$$

where $\Delta P^* = \Delta P/\Delta P_0$.

The baseline value of intraocular pressure (IOP), represented by P_0, is related to the baseline outflow Q_0 according $P_0 = Q_0\,(R_0 + R_d) + P_e$, per Equation 7 of the main text. With this, we define a dimensionless IOP as $P^* = (P - P_e)/(P_0 - P_e)$. Equation 7 of the main text can then be re-written as

$$P^* = Q^*\,(R^*(1 - R_d^*) + R_d^*) \quad \text{Eq. S2.6}$$

where $R_d^* = R_d/(R_0 + R_d)$ is the relative magnitude of distal to total outflow resistance at baseline.

Finally, we recognize from Equation 5 of the main text that h_0 is related to the resting SC height h_r according to $h_0^* = \exp(-\Delta P_0/E_0)$, where $h_0^* = h_0/h_r$ is a dimensionless parameter that represents the height of SC at baseline, when $\Delta P = \Delta P_0$ and $E = E_0$, relative to the resting SC height when $\Delta P = 0$. We can now re-write Equation 5 from the main text as

$$h^* = h_0^{*\left(\frac{\Delta P^*}{E^*} - 1\right)} \quad \text{Eq. S2.7}$$

where $h^* = h/h_0$ is the dimensionless height of SC relative to the baseline condition.

Together, Equations S2.1-S2.7 provide 7 relationships for 8 unknown dimensionless variables ($C^*,\ \tau^*,\ R^*,\ E^*,\ h^*,\ Q^*,\ \Delta P^*$, and P^*), corresponding to Equations 1*-7* of the main text. The one additional relationship needed to constrain the system is given by knowledge of the system state (constant pressure vs. constant flow) that defines either Q^* or P^* to be a fixed value, which is equal to unity if the perfusion is performed at the baseline state. Table 1 of the main text summarizes the dimensionless variables and parameters.
