# Supplementary material for: The Factors Affecting the Stability of IOP Homeostasis
Source: Invest Ophthalmol Vis Sci. 2024 Jun 4;65(6):4. doi: 10.1167/iovs.65.6.4 (PMC11157970; doi:10.1167/iovs.65.6.4)
Supplement: Supplement 3 [file iovs-65-6-4_s003.pdf]

The Time-Varying Response of the Outflow System to Small Perturbations

Supplemental Information 3 to

The Factors Affecting the Stability of IOP Homeostasis

Darryl R. Overby[1], C. Ross Ethier[2], Changxu Miao[1], Ruth A. Kelly[3], Ester Reina-Torres[1], W. Daniel Stamer[3]

In this supplemental information, we examine how the outflow system changes over time in response to small perturbations from baseline. These results complement the stability analysis presented in the main text, showing that the outflow system is more prone to instability under constant pressure relative to constant flow perfusion.

Numerical Considerations

In our analysis, we examine computationally how the outflow system changes over time in response to a small perturbation from baseline. As a perturbation, we impose a small non-zero change (positive or negative) in the dimensionless flow rate or pressure that persists for a brief time interval. This approximates the idealized scenario, where the eye at baseline experiences transient perturbations or noise that push the eye away from baseline. Such perturbations could arise due to instantaneous pressure spikes associated with blinks or saccades, as occur under normal physiological conditions *in vivo*[1,2], or due to disturbances in the flow or pressure expected for any experimental perfusion system. We aimed to determine

whether such a perturbation caused the system to return towards baseline, indicating a stable equilibrium, or whether the system diverged from baseline, indicating an unstable equilibrium.

To calculate how the system evolves over time, we solve the dimensionless governing equations (Equations 1*-7* of the main text) using the ode45 function in MATLAB (R2022a) applying the initial condition that $C^* = 1$. The equations are solved from $t^* = -1$ to 10 using an adjustable time step to achieve a defined relative tolerance of 10^{-13} and absolute tolerance of 10^{-14} in C^*. The perturbation is applied as step function to Q^* or P^*, changing discontinuously from 1 to $1 + \varepsilon_{Q^*/P^*}$ at $t^* = 0$, where ε_{Q^*/P^*} is the perturbation magnitude under constant flow or pressure, and then discontinuously back to 1 at $t^* = 0.1$, maintaining a value of unity for all future times. We explore both positive and negative perturbations, setting $\varepsilon_{P^*} = \pm 0.01$ and $\varepsilon_{Q^*} = \pm 0.10$. The larger value of ε_{Q^*} was by design: since constant flow condition is more stable than constant pressure, we tested the system's stability using a larger perturbation in constant flow mode. The MATLAB code used to produce the temporal results is given in the Appendix.

Results

The outflow system begins at its baseline state, which is a steady-state equilibrium because $\frac{d^*C}{dt^*} = 0$ whenever all dimensionless variables are equal to unity in Equation 3*. At time $t^* = 0$, we apply a brief step perturbation that moves the system away from baseline. Our goal is to identify whether the system returns to baseline or moves away from baseline in response to this perturbation, indicating a stable versus unstable equilibrium respectively.

We arbitrarily chose ρ^* and ξ^* to be either 0.5 or 1.5, and we fixed α^*, η^*, R_d^* and h_0^*. Setting ρ^* and ξ^* to a value of 0.5 represents the situation where outflow resistance and TM stiffness are moderately affected by NO, while a value of 1.5 represents a strong dependence on NO concentration (i.e., a perturbation in C imposes a 150% greater effect on outflow resistance or TM stiffness). The other parameter values were chosen to set shear-induced

NO production to roughly twice that of baseline ($\alpha^* = 2.0$), an equivalent second-order and first-order decay rate at baseline conditions ($\eta^* = 1.0$), distal resistance equal to one-quarter of total outflow resistance ($R_d^* = 0.25$), and the SC lumen at baseline to be narrowed by 40% relative to its resting height at zero pressure ($h_0^* = 0.60$). These parameters roughly approximate the human physiological state, as justified in the main text.

Constant Flow Perfusion

For constant flow perfusion, we consider a brief 10% increase or decrease ($\varepsilon_{Q^*} = \pm 0.10$) in the dimensionless flow rate Q^* applied at $t^* = 0$ that lasts until $t^* = 0.1$, after which time Q^* returns to a value of unity and remains there (Figure S3.1A). In response, the dimensionless intraocular pressure P^* exhibits an immediate change, similar in magnitude to that imposed on Q^*, followed by a decay in 3 of the 4 parameter sets, during which P^* and other parameters return to baseline within a few units of dimensionless time t^*, indicating stability (Figure S3.1B). In one case ($\rho^* = 0.5$ and $\xi^* = 1.5$, indicated by the orange tracings in Figure S3.1B), P^* does not return to baseline, indicating instability.

A similar trend is seen with all other dimensionless variables, with a consistent stable versus the single unstable response observed for all variables (Figure S3.1C-H). The single unstable case coincides with a time-dependent increase (or decrease, for the negative perturbation) in NO concentration C^*, which was associated with corresponding changes to the shear stress τ^* in SC and changes in the opposite direction to the height of SC lumen h^*, outflow resistance R^* and TM stiffness E^*. These results indicate that shear-mediated IOP homeostasis is largely stable, within limits, under conditions of constant flow perfusion.

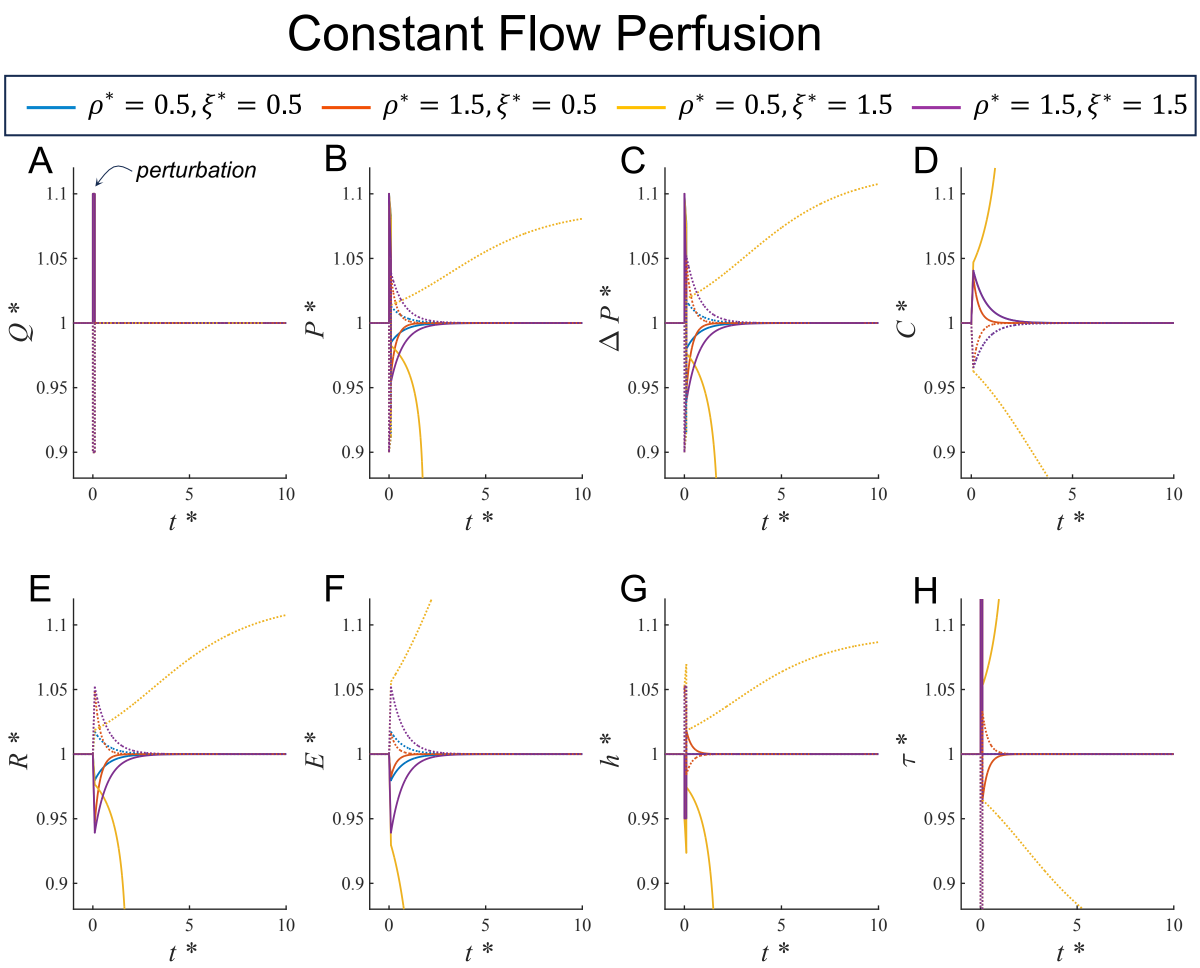

Figure S3.1: Temporal response of the model to a 10% perturbation in Q^* applied between $t^* = 0.00$ and $t^* = 0.10$ for the case of otherwise constant flow perfusion. Tracings represent the effect of different values of ρ^* and ξ^* (either 0.5 or 1.5), while fixing $\alpha^* = 2.0, \eta^* = 1.0, R_d^* = 0.25, h_o^* = 0.60$, and $\varepsilon_{Q^*} = \pm 0.10$. Solid tracings represent positive perturbations in Q^* ($\varepsilon_{Q^*} = +0.10$), while dotted tracings represent negative perturbations in Q^* ($\varepsilon_{Q^*} = -0.10$). The system is stable in 3 of the 4 examined parameter sets, returning to a baseline value of unity in all cases except for $\rho^* = 0.5$ and $\xi^* = 1.5$.

Constant Pressure Perfusion

Next, we considered constant pressure perfusion, utilizing the same parameter values as for constant flow above except applying a smaller perturbation of 1% in P^* ($\varepsilon_{P^*} = \pm 0.01$; Figure S3.2B). As shown in Figure S3.2A, the perturbation introduces an immediate response in Q^*. However, in 3 of the 4 parameter sets, Q^* diverges continuously from baseline over time, and a similar response is observed for all other variables (Figure S3.2C-H).

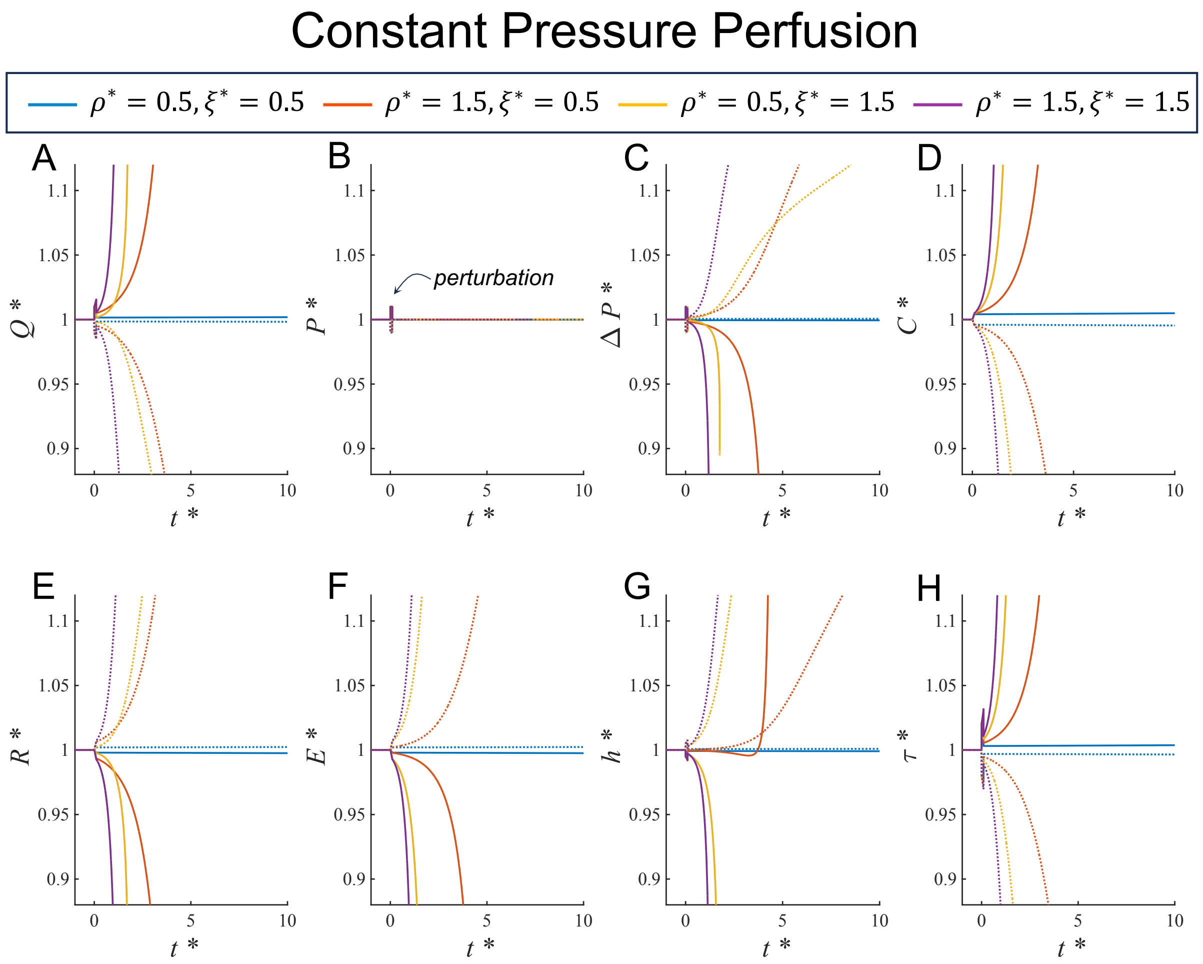

Figure S3.2: Temporal response of the model to a 1% perturbation in P^* applied between $t^* = 0.00$ and $t^* = 0.10$ for the case of constant pressure perfusion. Parameter values are the same as that used for Figure S3.1, setting ρ^* and ξ^* to either 0.5 or 1.5, while fixing $\alpha^* = 2.0$, $\eta^* = 1.0$, $R_d^* = 0.25, h_o^* = 0.60$, and $\varepsilon_{P^*} = \pm 0.01$. Solid tracings represent positive perturbations in P^* ($\varepsilon_{P^*} = +0.01$), while dotted tracings represent negative perturbations in P^* ($\varepsilon_{P^*} = -0.01$). The system is stable in only 1 of the 4 examined parameter sets, diverging from baseline in all cases except for $\rho^* = 0.5$ and $\xi^* = 0.5$.

The single case that exhibits stability for constant pressure ($\rho^* = 0.5$ and $\xi^* = 0.5$, indicated by the blue tracings in Figure S3.2) quickly returns to baseline, or near baseline, for all variables. This same parameter set also exhibits stability for constant flow, revealing that some parameter sets are always stable regardless of perfusion conditions. In contrast, of the 3 parameter sets that exhibit instability under constant pressure, only 1 of these sets also exhibits instability under constant flow. This reveals that some parameter sets are always

unstable, regardless of whether the perfusion is performed at constant pressure or constant flow. Further, and more importantly, there exists a range of parameter values where the system is stable under constant flow but unstable under constant pressure. These results demonstrate that the stability of shear-mediated IOP homeostasis depends on whether the outflow system is perfused under constant flow versus constant pressure, with the system appearing more stable under constant flow.

Comparison Against Predictions from Stability Analysis

Next, we compare how the time-dependent responses shown in Figures S3.1 and S3.2 compare against the stability criteria predicted by Equations 9 and 10 of the main text. Figure S3.3 shows the domains of stability and instability in the parameter space of ρ^* and ξ^*, with the other dimensionless parameters assigned as $R_d^* = 0.25$, $\alpha^* = 2.0$, $\eta^* = 1.0$ and $h_0^* = 0.6$ to match the situations shown in Figures S3.1 and S3.2. For constant flow perfusion (Figure S3.3A), the domain of instability (indicated in blue) is confined to the upper left of the parameter space, while most of the parameter space exhibits stability (indicated in yellow). The red line represents the critical value of $\xi_{c,Q}^*$ predicted by Equation 9 of the main text, above which the baseline state becomes unstable. The data points indicated in Figure S3.3A represent the 4 cases examined in Figure S3.1. One of these cases ($\rho^* = 0.5, \xi^* = 1.5$) lies in the unstable domain, matching the one unstable case observed in Figure S3.1 (orange tracing). The remaining 3 data points match the three stable cases observed in Figure S3.1.

For constant pressure perfusion (Figure S3.3B), the domain of instability (blue region) is larger relative to that for constant flow perfusion. Further, for all $\rho^* > 0$, the value of $\xi_{c,P}^*$ (indicated by the red line in Figure S3.3B) is smaller than the corresponding value of $\xi_{c,Q}^*$ (red line in Figure S3.3A). Three of the 4 data points lie deep within the unstable domain, matching the three unstable cases observed in Figure S3.2. The single case that appears stable in Figure S3.2 (blue tracing) corresponding to $\rho^* = 0.5$ and $\xi^* = 0.5$ lies just above the predicted critical value of $\xi_{c,P}^* = 0.492$, and is thus actually unstable with respect to baseline. Consistent

with this, close examination of Figure S3.2 shows that the blue tracings do not stabilize exactly back to baseline following the perturbation but converge to a second equilibrium that is slightly above or below unity. These results demonstrate that the baseline state is more prone to instability under constant pressure perfusion, relative to constant flow. Further, the predictions from the stability analysis given by Equations 9 and 10 match the temporal response of the system following small perturbations in Q^* or P^*.

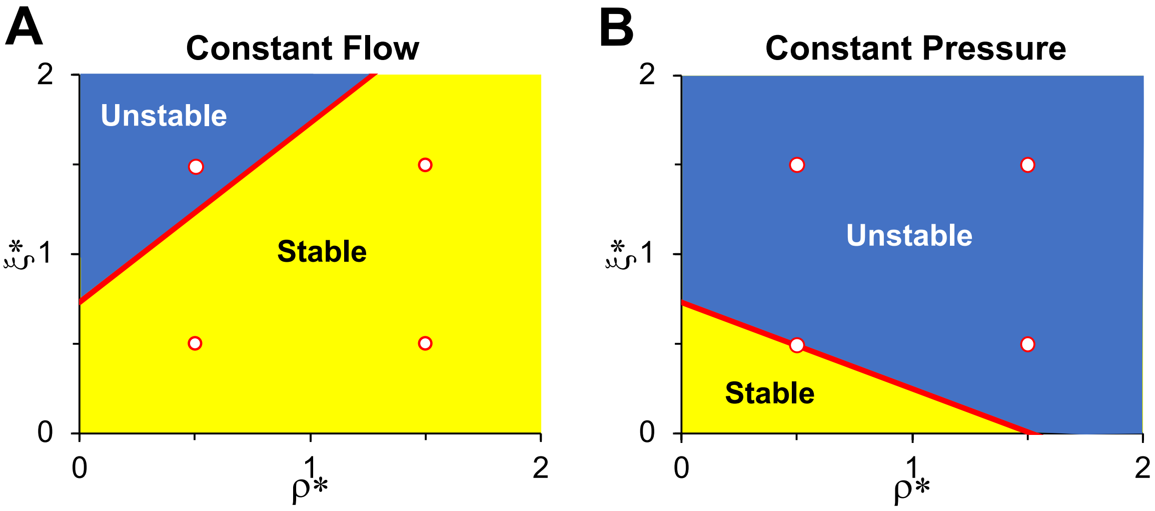

Figure S3.3: Domains of stability (yellow) and instability (blue) in the parameter space of ρ^* and ξ^* under constant flow (A) and constant pressure (B) perfusion. The four data points correspond to the cases plotted in Figures S3.1 and S3.2. The red lines are the predictions of $\xi^*_{c,Q}$ and $\xi^*_{c,P}$ from Equations 9 and 10, respectively, from the main text. The other dimensionless parameters are $\alpha^* = 2.0$, $\eta^* = 1.0$, $R_d^* = 0.25$ and $h_0^* = 0.6$. Note that the point located at $\rho^* = 0.5$ and $\xi^* = 0.5$ in panel B lies just above the stability threshold of $\xi^*_{c,P} = 0.492$.

As described in the main text, the regions of stability are defined based on the slope of $\frac{d}{dC^*}\left(\frac{dC^*}{dt^*}\right)$. Wherever $\frac{d}{dC^*}\left(\frac{dC^*}{dt^*}\right) > 0$, then a perturbation that increases C^* would correspond to a positive value of $\frac{dC^*}{dt^*}$, which in turn would further increase C^*, and lead to a divergence from baseline, indicating instability. In contrast, wherever $\frac{d}{dC^*}\left(\frac{dC^*}{dt^*}\right) < 0$ then a perturbation that increases C^* yields a negative value of $\frac{dC^*}{dt^*}$ that opposes the perturbation and tends to return the system towards baseline, implying stability. As illustrated in Figures S3.4 and S3.5, points

that lie within the stable domain have a negative slope when plotting $\frac{dC^*}{dt^*}$ vs C^*, while points lying within the unstable domain have a positive slope, in agreement with the stability criterion.

In some cases, it is apparent that a second equilibrium exists apart from the baseline state. For example, point 1 in Figure S3.4, corresponding to $\rho^* = 0.5$ and $\xi^* = 1.5$ under constant flow perfusion, where a second equilibrium exists near $C^* \approx 0.75$, which is a stable equilibrium with a negative slope. For this case, in response to a negative perturbation applied at baseline, the system converges to this second equilibrium state (see orange dotted tracings in Figure S3.1). However, in response to a positive perturbation, the deviation grows continuously because there is no second equilibrium for $C^* > 1$ (see solid orange tracings in Figure S3.1). The other secondary equilibria seen in Figure S3.4 (e.g., points 2 and 4) are unstable. Because our model is limited to the baseline state and small perturbations about baseline, our model is unable to reliably predict the existence of additional stable states removed from the baseline state, where non-linearities or saturation may become important. For this reason, we restrict our analysis to the stability at the baseline state, where the model is valid, and avoid predictions of secondary equilibria.

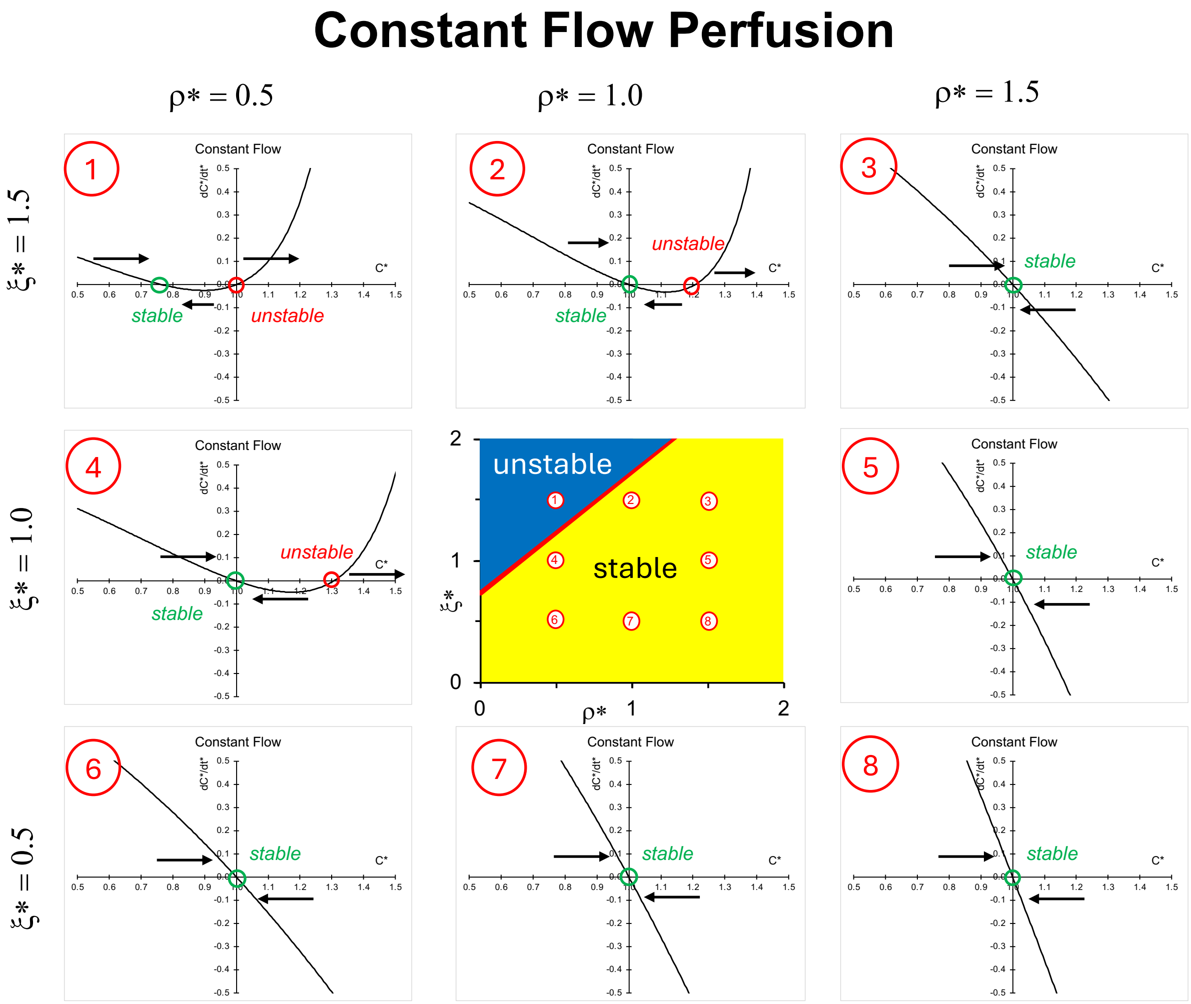

Figure S3.4: Plots of the relationship between dC^*/dt^* and C^* for selected points within the domain of ρ^* and ξ^* for constant flow perfusion, where the parameters are chosen to match Figures S3.1 and S3.3 ($\alpha^* = 2.0$, $\eta^* = 1.0$, $R_d^* = 0.25$, $h_0^* = 0.6$). In the stable domain (yellow), the selected points all have negative slopes of dC^*/dt^* versus C^* at baseline, while in the unstable domain, the slopes are positive at baseline, consistent with the stability criterion. Circled numbers in red indicate the location of that point within the domain of (ρ^*, ξ^*). Green circles indicate stable equilibria and small red circles indicate unstable equilibria. Black arrows indicate the direction that the system will move in response to a perturbation from equilibrium.

Constant Pressure Perfusion

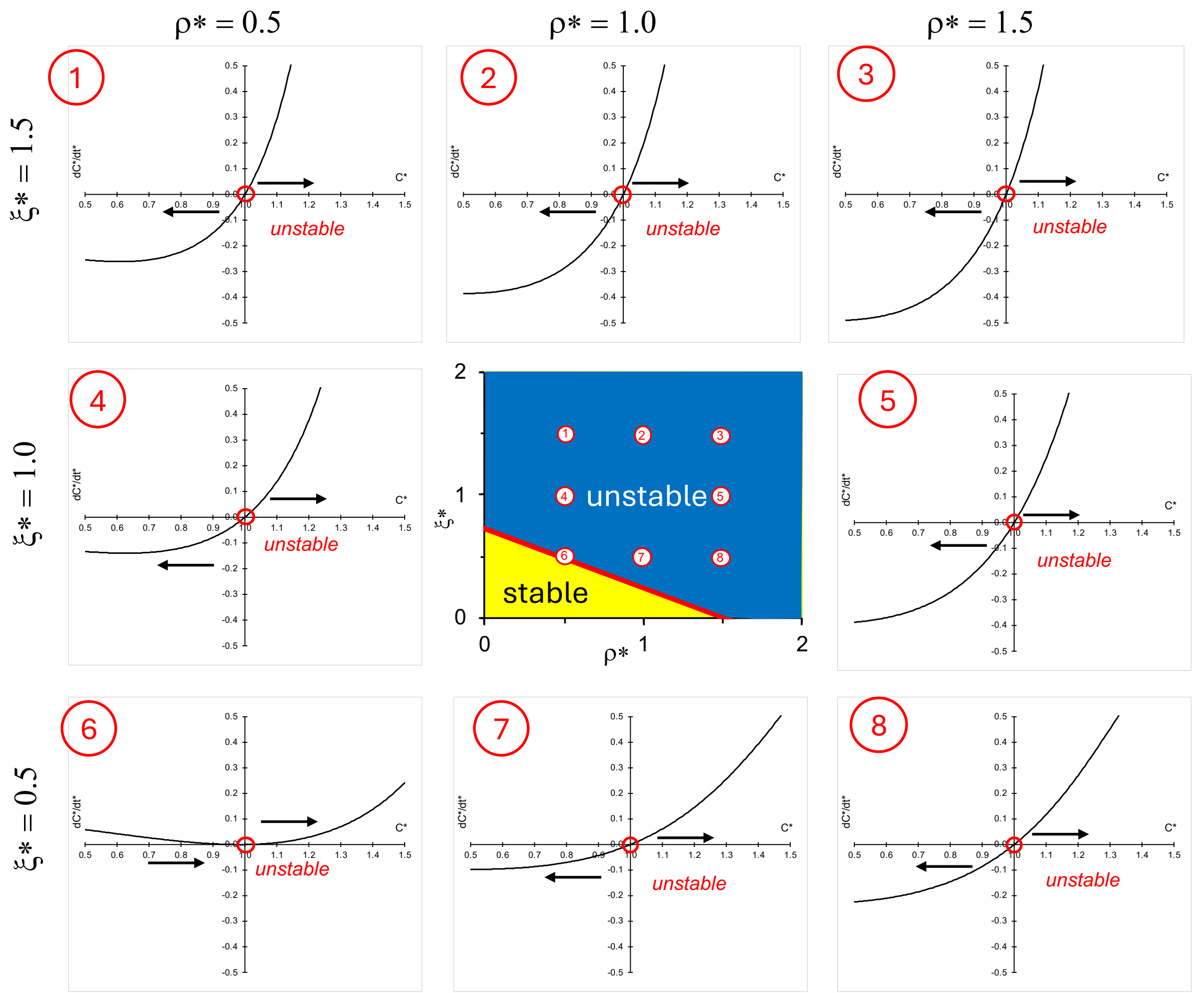

Figure S3.5: Plots of the relationship between dC^*/dt^* and C^* for selected points within the domain of ρ^* and ξ^* for constant pressure perfusion, where the parameters are chosen to match Figures S3.2 and S3.3 ($\alpha^* = 2.0$, $\eta^* = 1.0$, $R_d^* = 0.25$, $h_0^* = 0.6$). All selected points fall within the unstable domain (blue) and with positive slopes of dC^*/dt^* versus C^* at baseline, consistent with the stability criterion. Circled numbers in red indicate the location of that point within the domain of (ρ^*, ξ^*). Green circles indicate stable equilibria and small red circles indicate unstable equilibria. Black arrows indicate the direction that the system will move in response to a perturbation from equilibrium.

References

1. Coleman, D. J. & Trokel, S. Direct-recorded intraocular pressure variations in a human subject. *Arch. Ophthalmol.* **82**, 637–640 (1969).

2. Downs, J. C. IOP telemetry in the nonhuman primate. *Exp. Eye Res.* **141**, 91–98 (2015).

Appendix

The Matlab code pasted below was used to solve Equations 1*-7* of the main text and to produce the time-dependent results shown in Figures S3.1 and S3.2. This code runs successfully on Matlab version R2022a.

```
% Sept 06, 2023, Darryl Overby, Imperial College London (C)
% Revised July 24, Sept 11-12, Nov 22, 2022, May 15, 2023

% This code calculates the time-dependent response of the outflow system following a
% step perturbation in either flow or pressure. For simplicity, we do not include the code used
to generate Figures S3.1 & S3.2, but these follow standard procedures once the results are
extracted from the solution array Sol{i}. See last few lines of the code to understand the
structure of Sol and how to extract the necessary data.

%% initialise and define parameters

clear all;close all;clc;

% define state condition
% flag = 0;   % constant flow
 flag = 1;    % constant pressure

% Define parameters for system; all lists must be the same length
Rd_star_list =      [0.25,0.25,0.25,0.25,0.25,0.25,0.25,0.25];  % ratio of distal to total
resistance at baseline
ho_star_list =      [0.60,0.60,0.60,0.60,0.60,0.60,0.60,0.60];  % fraction of SC open at
baseline (1 - fractional collapse)
alpha_star_list =   [2.00,2.00,2.00,2.00,2.00,2.00,2.00,2.00];  % dimensionless gain of
shear-induced NO production
eta_star_list =     [1.00,1.00,1.00,1.00,1.00,1.00,1.00,1.00];  % dimensionless ratio of 2nd
to 1st order NO decay
rho_star_list =     [0.50,1.50,0.50,1.50,0.50,1.50,0.50,1.50];  % relative change in IW
resistance per unit change in NO
xi_star_list =      [0.50,0.50,1.50,1.50,0.50,0.50,1.50,1.50];  % relative change in TM
stiffness per unit change in NO


% define pertubation
if flag == 0    % constant flow
    eQP_star_list = [0.10,0.10,0.10,0.10,-0.10,-0.10,-0.10,-0.10];
elseif flag == 1    % constant pressure
    eQP_star_list = [0.01,0.01,0.01,0.01,-0.01,-0.01,-0.01,-0.01];
end


% Define input parameters for numerical solution
tmin = -1.0;    % dimensionless start time
tmax = 11.0;    % dimensionless end time
c0 = 1.00;      % initial dimensionless value of Cstar

% Define start and stopping time for the perturbation
eQP_start = 0.00;   % starting time for perturbation
eQP_stop  = 0.10;   % ending time for perturbation

% calculated parameters
```

```
num = length(Rd_star_list);      % the number of parameter sets

%% Define function handles
% for simplicity, we define the following variables
% t = t_star
% c = C_star
% a = alpha_star
% n = eta_star
% p = rho_star
% x = Xi_star
% r = Rd_star
% h = h0_star
% v = v_star
% e = eQP_star

% define anonymous function handles
R_star = @(t,c,a,n,p,x,r,h,e) 1 - p*(c-1);   % Eq 10
E_star = @(t,c,a,n,p,x,r,h,e) 1 - x*(c-1);   % Eq 2*

if flag == 0    % constant flow

    Q_star = @(t,c,a,n,p,x,r,h,e) 1.0 + ...
        e * (t >= eQP_start) .* (t < eQP_stop);    % add perturbation;

    P_star = @(t,c,a,n,p,x,r,h,e) Q_star(t,c,a,n,p,x,r,h,e) .* ...
                        (R_star(t,c,a,n,p,x,r,h,e) * (1-r) + r);        % Eq 7*
elseif flag == 1    % constant pressure

    P_star = @(t,c,a,n,p,x,r,h,e) 1.0 + ...
        e * (t >= eQP_start) .* (t < eQP_stop);    % add perturbation

    Q_star = @(t,c,a,n,p,x,r,h,e) P_star(t,c,a,n,p,x,r,h,e) ./ ...
                ( R_star(t,c,a,n,p,x,r,h,e) .* (1-r) + r);             % Eq 7*
end

dP_star = @(t,c,a,n,p,x,r,h,e) ...
            Q_star(t,c,a,n,p,x,r,h,e) .* R_star(t,c,a,n,p,x,r,h,e);     % Eq 6*

h_star = @(t,c,a,n,p,x,r,h,e) ...
    h .^ (dP_star(t,c,a,n,p,x,r,h,e) ./ E_star(t,c,a,n,p,x,r,h,e) - 1); % Eq 5*

tau_star = @(t,c,a,n,p,x,r,h,e) ...
          Q_star(t,c,a,n,p,x,r,h,e) ./ (h_star(t,c,a,n,p,x,r,h,e).^2);  % Eq 4*

%% iterate through each parameter set and solve ODE

for i = 1:num

    % define parameters for current set
    h = ho_star_list(i);
    a = alpha_star_list(i);
    n = eta_star_list(i);
    r = Rd_star_list(i);
    p = rho_star_list(i);
    x = xi_star_list(i);
    e = eQP_star_list(i);

    % solve time-dependent ODE for c
    opts = odeset('Reltol',1e-13,'AbsTol',1e-14,'Stats','on');
    [t_sol,c_sol] = ode45(@(t,c) a .* (tau_star(t,c,a,n,p,x,r,h,e) - 1) ...
        - c/(1+n) - (n/(1+n)).*(c.^2) + 1, [tmin tmax], c0, opts);      % Eq 3*

    % integrate Q_star to determine perfused volume
    Q_sol = Q_star(t_sol,c_sol,a,n,p,x,r,h,e);
    v_sol = cumtrapz(t_sol, Q_sol);
                % dV*/dt* = Q*; Vo = Qo*time scale

    % save solution for all relevant variables in a cell array
    Sol{i} = [t_sol, c_sol, ...
     Q_star(t_sol,c_sol,a,n,p,x,r,h,e), P_star(t_sol,c_sol,a,n,p,x,r,h,e),...
     dP_star(t_sol,c_sol,a,n,p,x,r,h,e), h_star(t_sol,c_sol,a,n,p,x,r,h,e),...
     tau_star(t_sol,c_sol,a,n,p,x,r,h,e), R_star(t_sol,c_sol,a,n,p,x,r,h,e), ...
     E_star(t_sol,c_sol,a,n,p,x,r,h,e), v_sol];
end
```
