# Supplementary material for: The Factors Affecting the Stability of IOP Homeostasis
Source: Invest Ophthalmol Vis Sci. 2024 Jun 4;65(6):4. doi: 10.1167/iovs.65.6.4 (PMC11157970; doi:10.1167/iovs.65.6.4)
Supplement: Supplement 4 [file iovs-65-6-4_s004.pdf]

The Factors Affecting the Stability of IOP Homeostasis

Darryl R. Overby, C. Ross Ethier, Changxu Miao, Ruth A. Kelly, Ester Reina-Torres, W. Daniel Stamer

Table of Dimensional Variables and Parameters

Variables	Definitions	SI Units	Relevant Equations
R	Outflow resistance of JCT/IW	Pa s/m^3	Eq. 1
E	TM stiffness	Pa	Eq. 2
C	NO concentration along SC	kmol/m^3	Eq. 3
τ	Shear stress acting on SC cells	Pa	Eq. 4
h	Height of SC	m	Eq. 5
ΔP	Pressure drop across inner wall	Pa	Eq. 6
P	Intraocular pressure	Pa	Eq. 7 or fixed†
Q	Conventional outflow rate	m^3/s	Eq. 7 or fixed†
q	Characteristic flow rate in SC	m^3/s	$q = Q/4N$

Independent Parameters			
P_0	Baseline value of P at constant pressure	Pa	Only one of P_0 or Q_0 can be independent†
Q_0	Baseline value of Q at constant flow	m^3/s	
ρ	Sensitivity of R to changes in C	Pa s/kmol	Eq. 1
R_0	Baseline value of R	Pa s/m^3	Eq. 1
ξ	Sensitivity of E to changes in C	Pa m^3/kmol	Eq. 2
E_0	Baseline value of E	Pa	Eq. 2
α	Shear-dependent production rate of NO	kmol/(Pa s m^3)	Eq. 3
γ	baseline production rate of NO	kmol/(s m^3)	Eq. 3
β_1	1st order reactive decay coefficient for NO	1/s	Eq. 3
β_2	2nd order reactive decay coefficient for NO	m^3/(kmol s)	Eq. 3
μ	Viscosity of aqueous humour	Pa s	Eq. 4
w	Anterior-posterior SC width	m	Eq. 4
h_r	Resting zero-pressure value of h	m	Eq. 5
P_e	Episcleral venous pressure	Pa	Eq. 7
R_d	Distal outflow resistance	Pa s/m^3	Eq. 7
N	Number of collector channels	--	$q = Q/4N$

Dependent Parameters			
P_0	Baseline value of P at constant flow	Pa	Eq. 7b if Q_0 is fixed†
Q_0	Baseline value of Q at constant pressure	m^3/s	Eq. 7c if P_0 is fixed†
τ_0	Baseline value of τ	Pa	Eq. 4b
h_0	Baseline value of h	m	Eq. 5b
C_0	Baseline value of C	kmol/m^3	Eq. 3b

† Of P and Q, one is a variable and the other is a fixed parameter. Specifically, P is a variable under constant flow perfusion when $Q = Q_0$, while Q is a variable under constant pressure perfusion when $P = P_0$.
